# Barriers and facilitators for pharmacist-led vaccination services: A systematic review using the Consolidated Framework for Implementation Research (CFIR)

**DOI:** 10.1016/j.rcsop.2025.100687

**Published:** 2025-11-25

**Authors:** Meral Abdulselam, Alla El-Awaisi, Ziad G. Nasr, Shahd Shaar, Maguy Saffouh El Hajj

**Affiliations:** a College of Pharmacy, QU Health Sector, Qatar University, Doha, Qatar; b Clinical Pharmacy and Practice Department, College of Pharmacy, QU Health Sector, Qatar University, Doha, Qatar; c Communicable Disease Center, Hamad Medical Corporation, Qatar

**Keywords:** Barrier, Facilitator, Pharmacist, Vaccination

## Abstract

**Background:**

Pharmacists are well positioned to contribute to the expansion of vaccination program outreach, owing to their high accessibility, pharmacotherapy knowledge, and patient-centered services. Over the past decade, the scope of pharmacy practice has progressively evolved, shifting from a product-oriented role toward more direct involvement in public health initiatives, including immunization. Despite this advancement, the full potential of pharmacists as immunization providers remains underutilized due to several barriers.

**Objective:**

This systematic review aims to synthesize evidence on the barriers and facilitators of pharmacist-led vaccination services using the Consolidated Framework for Implementation Research (CFIR).

**Method:**

A comprehensive literature search was conducted across multiple databases (Ovid/Medline, EMBASE, ISI Web of Science, ProQuest Dissertations, PROSPERO, Cumulative Index to Nursing and Allied Health Literature (CINAHL), Elsevier Science Direct, Health Management Information Consortium (HMIC), and Google Scholar. Studies were included if they focused on pharmacist-initiated vaccinations and reported relevant barriers or facilitators. The findings were mapped to CFIR.

**Results:**

A total of 33 studies were included. Barriers to pharmacist-led vaccination services were reported in 32 studies. The main barriers in the Outer Domain were mainly fear of conflicting roles with physicians and public attitudes, lack of physician support, patient unwillingness to receive vaccinations in community settings, liability and regulation related issues, and reimbursement for pharmacist vaccination services. In the Inner Domain, lack of pharmacist training and lack of adequate facilities for delivering community pharmacy vaccination services were the main barriers. Facilitators in the Outer Domain were professional recognition, cooperation between pharmacists and healthcare centers, and financial remuneration. In the Inner Domain, immunization training was frequently considered as a crucial element. Under the Individual Characteristics Domain, opportunities to strengthen the pharmacist–patient relationship, pharmacist interest, patient trust, and patient demand were considered as key facilitators.

**Conclusion:**

This study identified the main barriers and facilitators to pharmacist-led vaccination services using the CFIR framework, primarily within the Outer and Inner Setting domains. Findings highlight the need for targeted training, regulatory support, and reorganized reimbursement. Future research should explore context-specific interventions and evaluate training effectiveness to advance pharmacist-led immunization.

## 1 Introduction

Vaccination remains one of the most impactful public health initiatives, currently preventing 3.5–5 million deaths annually by protecting against infectious diseases and reducing at least 20 life-threatening infectious diseases.^1^ However, vaccination coverage remains inadequate globally.^2^ In 2022, the World Health Organization (WHO) reported that more than 21.9 million children missed their first routine measles immunization, while 14.3 million children did not receive the diphtheria vaccine due to limited access to immunization facilities.^2^ Additionally, it was reported that vaccination coverage among adults does not meet recommended target levels in nearly all countries, leading to occurrences of preventable diseases and disabilities.^3^ For instance, in 2019, the United States of America (USA) reported that only 21.8 % of adults received age-appropriate immunizations, including tetanus and diphtheria (Td), tetanus, diphtheria, and pertussis (Tdap), and influenza vaccines.^4^ Additionally, in 2022, the USA reported shingles vaccination coverage rates of 31.6 % highlighting challenges in achieving optimal vaccination rates.^5^

Pharmacists, due to their easy accessibility, medication-related expertise, and patient-centered approach, are well-positioned to expand vaccination program outreach6., 7..^8^ Over the last decade, their role has evolved beyond product dispensing to being actively involved in immunization efforts.^9^ For instance, several countries such as USA, Argentina, and the United Kingdom (UK) have embraced the role of pharmacists in vaccine administration^10^.^11^ Another review that primarily focused on the USA, Canada, Puerto Rico, and Japan indicated that pharmacists serving as immunizers, advocates, or both significantly raised immunization rates.^12^

However, the implementation of pharmacist-led vaccination services remains limited, particularly in low- and middle-income countries.^10^ Barriers include inadequate training, lack of knowledge regarding vaccines, governmental restrictions on pharmacy practice, and public concerns about pharmacists' ability to provide vaccination services.^13^ A study conducted in Lebanon found a high level of willingness among pharmacists to provide vaccination services; however, they reported legal and regulatory barriers for offering these services.^14^ Additionally, in Jordan, a significant percentage of the public expressed doubts about the readiness of pharmacies to offer vaccination services.^15^ While in Austria, legal liability and appropriate training were the most critical barriers for pharmacists to provide vaccination services.^16^

A previous systematic review assessed the barriers and facilitators affecting pharmacists' ability to act as immunizers using the (capability (C), opportunity (O) and motivation (M)- behavior (B) model).^17^ Barriers were identified in 26 studies across various domains including physical (training) and psychological (knowledge) capability, social opportunity (staff support, and reflective motivation (cost). Nonetheless, the majority of these studies were conducted in the US and Canada, with a few studies from other countries, thereby limiting the generalizability of its findings on current vaccination practices.^17^ Another systematic review, which included studies up to June 2016, found that pharmacy-based immunization services are not only feasible and well-accepted but also effective in improving vaccination rates among adults.^18^ State regulatory changes and the certification of pharmacists as immunizers have facilitated these outcomes but political and organizational barriers pose a further constraint to these services.^18^ Given that the review primarily targeted USA studies, it may not fully reflect the global status of current pharmacists-led vaccination services. Moreover, while the study explores the accessibility and feasibility of community pharmacies as vaccination sites, it does not address the barriers and facilitators associated with the delivery of pharmacist-led vaccination services.^18^

Recognizing the evolving global landscape of vaccination services, there is a need to examine both the barriers and facilitators of pharmacist-led vaccination services and to include studies from diverse regions to provide a more comprehensive perspective. This systematic review addressed this gap by using the Consolidated Framework for Implementation Research (CFIR) to guide our approach.^19^ This systematic review aimed to synthesize global evidence about barriers and facilitators to pharmacist-led vaccination services from multiple perspectives (public, patients, pharmacists, other health professionals, stakeholders, and policymakers) using the CFIR framework.^19^

Scoping reviews play an important role in identifying knowledge deficiencies in a field, but do not assess study quality or provide syntheses suitable for informing action or making decisions^20^.^21^ In contrast, the review objectives were confirmatory and more focused: to compile all eligible studies examining barriers and facilitators to pharmacist-initiated vaccination, critically assess these studies, and synthesize their findings within the CFIR to derive practical and policy related implications. These aims align more closely with the purpose of systematic reviews, which are designed to answer specific research questions, rather than with exploratory scoping reviews.^22^ Accordingly, we applied pre-defined eligibility criteria, extensive literature retrieval, duplicate screening, critical appraisal, and clearly documented synthesis processes, and subsequently aligned the review results to the CFIR to improve interpretability.^19^

## 2 Methods

### 2.1 Framework

The Consolidated Framework for Implementation Research (CFIR) was used in this review to identify and evaluate the barriers and facilitators to pharmacist-led vaccination services.^19^ The CFIR identifies and addresses pertinent issues that may affect the implementation and effectiveness of interventions^23^.^24^ Given the scope of the CFIR, it was considered suitable for addressing the complex and multilayered barriers faced in the implementation of vaccination programs in the pharmacy contexts [13].

In this study, we systematically referred to the official CFIR usage guide, which provides clear definitions for each of the five domains and their associated constructs, to ensure that contextual, organizational, and individual-level factors are accurately mapped to their respective domains and constructs.^25^

These barriers can range from individual pharmacist beliefs and knowledge (characteristics of individuals) to organizational and systemic challenges (inner and outer settings). Furthermore, CFIR's focus on the implementation process provides a detailed pathway for assessing the effectiveness of strategies used to engage stakeholders and overcome identified barriers.^19^ One of CFIR's strengths is its flexibility and adaptability across different health interventions and settings, making it particularly useful when analyzing diverse pharmacy environments globally, each with its own unique challenges and facilitators. Moreover, recent iterations of CFIR have incorporated elements from other models, such as the COM-B model,^26^ to further enrich its analytical capacity. These integrations enhance CFIR by ensuring that all relevant aspects of behavior change, and intervention effectiveness are considered offering a rich and detailed analysis.^19^

#### 2.1.1 Protocol and registration

This review was conducted in accordance with (Preferred Reporting Items for Systematic Reviews and Meta-Analyses).^27^ The protocol was registered and made available on PROSPERO at the Centre for Reviews and Dissemination (CRD42024496594).

#### 2.1.2 Data sources and search strategy

A comprehensive search process was conducted using various databases and search engines: Ovid/Medline, EMBASE, ISI Web of Science, ProQuest Dissertations, PROSPERO, Cumulative Index to Nursing and Allied Health Literature (CINAHL), Elsevier ScienceDirect, Health Management Information Consortium (HMIC), and Google Scholar. A manual search of reference lists from included studies was also performed to identify additional relevant literature.

Search terms were selected from various categories using PICO criteria pertaining to population (pharmacists) intervention (pharmacist-led vaccination services) comparator (Not applicable) and outcomes (barriers and facilitators).^28^ The search strategy included specific search terms which were combined differently across categories using Boolean operators. Adjustments were made according to different databases. No restrictions were applied for this search in terms of language or publication date. Data sources were searched since inception till February 2024. Duplicates were removed using Endnote software^29^

#### 2.1.3 Eligibility criteria and selection of studies

Studies were included in this systematic review if they met the following criteria:

1) They focused explicitly on vaccination services provided by pharmacists,
2) They identified barriers and/or facilitators related to pharmacist-led vaccination services.

Reviews, letters, editorials, and commentaries were excluded. Various electronic databases and grey literature were also screened for relevant insights such as (government/health authority sites and national pharmacy associations).

Two reviewers independently screened the titles and abstracts using Rayyan® software,^30^ following a calibration exercise on 50 records to ensure consistency in applying the eligibility criteria. The reviewers were Meral Abdelsalam (MA), a Master of Pharmacy candidate, and MH, a Clinical Associate Professor. The same procedure was applied for full-text screening in Rayyan®, using the review inclusion and exclusion criteria. Blinded mode was enabled in Rayyan® until conflict resolution. Any discrepancies at any stage were resolved through discussion, with a third reviewer (the other study investigators: ZN, SS, or AE) providing adjudication when necessary. Abstracts of non-English articles were translated into English using Google Translate® to enable eligibility screening.

#### 2.1.4 Data extraction and quality assessment

Study quality was assessed independently by two reviewers from the research team (MA, MH, ZN, SS, and AE) using the *Crowe Critical Appraisal Tool* (CCAT).^31^ The tool was chosen because it enables a single, rigorous approach to appraising studies of diverse designs and facilitates direct comparisons across methodologies. Many systematic reviews have applied the CCAT for quality appraisal, further confirming its validity and reliability^32^.^33^ Disagreements in quality assessment were resolved through discussion or consultation with a third reviewer.

A structured data extraction form was designed, adhering to the critical reporting components outlined in the PRISMA-S statement for systematic reviews.^34^ The form encompassed various details including country, publication year, objectives, design, inclusion and exclusion criteria, primary outcomes in relation to barriers and facilitators for pharmacists-led vaccination services, study limitations, and other pertinent information. This form was pilot tested on five randomly selected studies from the included set and refined accordingly. Two investigators (MA, MH) independently extracted the data using this form, and their findings were collectively reviewed.

#### 2.1.5 Data synthesis and analysis

The studies' reported barriers and facilitators for pharmacist vaccination services from different perspectives (pharmacists, healthcare provides, public and stakeholders) were narratively synthesized and presented in alignment with the CFIR domains: Innovation, Outer Setting, Inner Setting and Individual Characteristics.^19^ The Implementation Process domain encompasses the concrete actions taken to establish interventions including planning, engaging, executing, and reflecting on the implementation activities.^19^ This domain remains a highly important component for understanding how pharmacist-led vaccination services might be operationalized especially concerning stakeholder and feedback pathways to sustain vaccinations. This domain was not included as the target of the current review was to identify barriers rather than providing detailed information about the procedural aspects of implementation especially in relation.

Two reviewers (MA and MH) independently coded the findings sections of each included study, consulting the discussion sections when necessary for clarification, using a pre-specified CFIR codebook.^35^ The codebook outlined definitions for each domain and construct, accompanied by decision rules and representative indicators. During an initial calibration exercise involving three studies, the reviewers compared coding outputs, refined construct rules, and finalized the codebook. Subsequently, all remaining studies were double-coded independently. Discrepancies related to construct selection, barrier/facilitator classification, or adequacy of supporting evidence were resolved through iterative discussion and constant comparison, with a third reviewer (ZN, SS, or AE) adjudicating unresolved cases. Multiple construct mappings were permitted; however, a primary construct was assigned based on established decision rules. Aggregation across constructs was deferred until completion of study-level coding.

## 3 Results

**Fig. 1:**
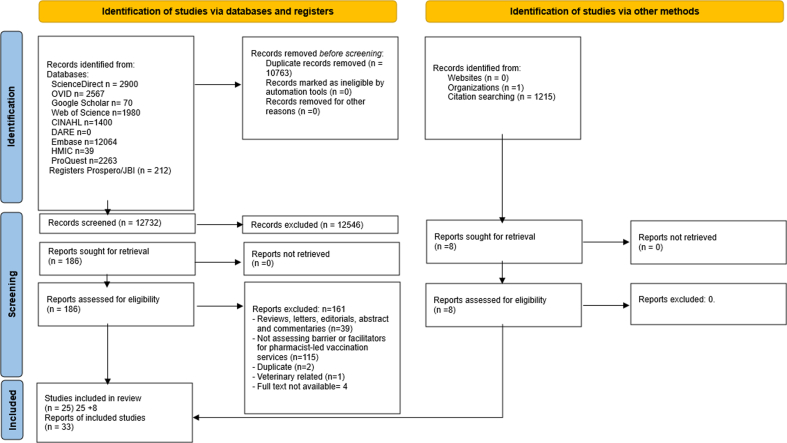
PRISMA diagram for article selection process. n = number of studies.

A total of 23,495 records were identified through searching the databases. After removal of duplicates, 12732 records were screened for potential inclusion in the review. At title and abstract stage, 12,546 records were excluded; 186 full-text articles were assessed for eligibility. Twenty-five records were considered eligible. Eight extra records were selected through the manual search. As a result, 33 records were included in the review. (Fig. 1).All articles were published in English.

### 3.1 Study characteristics

Table 1 outlines the studies' characteristics. Most studies were conducted in North America (*N* = 19),^36^, ^37^, ^38^, ^39^, ^40^, ^41^, ^42^, ^43^, ^44^, ^45^, ^46^, ^47^, ^48^, ^49^, ^50^, ^51^, ^52^, ^53^, ^54^ followed by the Middle East & North Africa (*N* = 6).^14^^,^^55^, ^56^, ^57^, ^58^, ^59^ One record is a global report published by the International Pharmaceutical Federation (FIP) on current pharmacy impact on immunization.^8^

**Table 1:** Studies characteristics and other relevant information.

Country	Author/ citation	Date of Data collection	Study Design	Data collection form	Personnel/N / Vaccination type	Study Objective(s)	Inclusion Criteria	Exclusion Criteria	Limitations
Argentina	Helena Rosado *et al.,*/^8^	February and March 2016.	Survey	Survey	137 FIP member organizations (pharmacists) / vaccination in general	To have an insight into the role of pharmacists in immunization across the world and the influence of these activities	FIP organization members	NM	NM
Australia									
Belgium									
Bolivia									
Brazil									
Canada									
China									
Congo (Dem. Rep.)									
Costa Rica									
Denmark									
Ecuador (Quito)									
Ethiopia									
Finland									
France									
Germany									
Hong Kong, China									
Hungary									
Iceland									
India									
Iraq									
Ireland									
Israel									
Italy									
Japan									
Jordan									
Lebanon									
Netherlands									
New Zealand									
Nigeria									
Norway									
Pakistan									
Paraguay (Asunción)									
Philippines									
Poland									
Portugal									
Russian Federation									
Senegal									
South Africa									
Spain									
Switzerland									
United Arab Emirates									
Ukraine									
United Kingdom									
United States of America									
Uruguay									


Country	Author/ citation	Date of Data collection	Study Design	Data collection form	Personnel/N / Vaccination type	Study Objective(s)	Inclusion Criteria	Exclusion Criteria	Limitations
Australia	H Laetitia Hattingh *et al.,* /^65^	Between March and October 2015	Mixed method	Data through surveys,	Pharmacies baseline surveys = 78 completed exit surveys; computer records = 57 immunizer pharmacists were interviewed= 25. /	To assess the uptake of Western Australian pharmacist vaccination services, and the facilitators and barriers experienced by	Of the pharmacies, 133 offered pharmacist vaccination services in 2015 and were eligible to participate in the study	Others were excluded	This study was done in WA thus findings might not be generalizable to other Australian jurisdictions due to slight differences in policies.
				pharmacy computer records and immunizer pharmacist	No vaccination specification	pharmacy staff			
				interviews		.			
Indonesia	Susi Ari Kristina *et al.,* /	March to April 2021.	Cross sectional study	Online survey	Pharmacists	To explore community pharmacists’ perceptions and detect reasons that influence their motivation to administer the COVID-19 vaccine	Pharmacists from community pharmacies.	NM	The results may not be representative for community pharmacists in a single nation.
	-60				N= 120 / COVID-19 vaccine				The survey was done online and independently, it had limited control over respondents.
		
Malaysia	Wei Chern Ang *et al.,*^61^	April to June 2021	Cross-sectional study	Survey	Community pharmacists	To identify possible barriers to and factors supporting the provision of vaccination by pharmacist in Malaysia.	Community pharmacies registered in Malaysia	Registered pharmacist working in community pharmacies.	Absence of applicant coverage in the rural areas.
					N= 492 /				
					No vaccination specification				
Austria	Nikolaus Lindner *et al.,* /^16^	NM	Cross-sectional	Survey	Community pharmacists	To identify the willingness of Austrian community pharmacists to administer immunizations in the future.	Community pharmacists	NM	Nature of a questionnaire study (response and selection bias).
					N= 380 /				The sole representation of the pharmacists’ perspective.
					No vaccination specification				
Poland	Piotr Merks *et al.,* /^62^	February and August 2020	Cross sectional-Survey	Survey	Pharmacists	To assess the willingness of	Closed pharmacist groups.	Others	The response rates are low.
					N= 1666/	community pharmacists following the Pharmacist in providing immunization services.			Findings might differ in the other areas and local pharmacy jurisdictions due to differences in vaccination service opinions in pharmacies.
					No vaccination specification				
		
		
		


Country	Author/ citation	Date of Data collection	Study Design	Data collection form	Personnel/N / vaccination type	Study Objective(s)	Inclusion Criteria	Exclusion Criteria	Limitations
UK	Adam Pattison *et al.,* /^64^	January and February 2021	Qualitative phenomenological study	Semi-structured interviews	Pharmacists	To identify pharmacists' roles and experiences in working within a COVID-19 vaccination program.	Pharmacists with experience working in a COVID-19 Vaccination Centre	10were excluded	Participants were enrolled from a single vaccination center.
					N= 34 / COVID-19 vaccine				The same investigator completed all the data collection.
Jordan	Mohammad B Nusair *et al.,* / A47^55^	October 2019 to December 2019	Qualitative Study: cross sectional exploratory	Semi-structured telephone interviews	Pharmacists	To discuss community pharmacists’ beliefs on	Pharmacists	NM	Small sample size
					N= 19 / influenza vaccine	providing influenza vaccine services in community pharmacies in Jordan.			Use of telephone interviews.
									Social desirability bias.
		
Lebanon	Dalal Youssef *et al.,.*/^14^	1 and 31st December 2020	Cross‑sectional study	Survey	Community pharmacists N=412 / No vaccination specification	To investigate Lebanese pharmacists’ willingness to develop their practice scope to include vaccine administration.	Community pharmacists working currently in pharmacy setting and who agreed to participate to the study were eligible for participation.	Clinical pharmacists, those who were out of the country at the time of the survey, as well as those not practicing.	The cross-sectional design of this study did not allow to infer causality.
									Depending on community pharmacists’ self-reported information, which makes it eligible to the disadvantages of desirability biases.
									Selection bias.

Lebanon	Fouad Sakr *et al.,* /^56^	NM	Cross-sectional	Survey	Pharmacists	To identify challenges possible chances to provide immunization in the Lebanese pharmacy practice.	All registered pharmacists in	Others were excluded	Cross‐sectional design that cannot assess temporality.
					N= 315 / No vaccination specification		Lebanon. Were considered eligible for participation.		Deterioration of financial conditions may be residual confounders to the cost of professional development of pharmacists.
									Response bias.
									Selection Bias due to snowball sampling.
		
Lebanon	Dalal Youssef *et al.,* /^57^	1st of November to the end of December 2020.	Web‑based, cross‑sectional study	Anonymous,	Community pharmacists	To assess the Lebanese CPs’ willingness to administer influenza vaccine.	Community pharmacists working currently in pharmacy setting were eligible for participation.	Clinical pharmacists, retired CPs, were out of the country at the time of the survey, and those who refused to participate in the study were also excluded.	Desirability biases.
				questionnaire	N = 412 / Influenza vaccine				Selection bias.
		


Country	Author/ citation	Date of Data collection	Study Design	Data collection form	Personnel/N / vaccination type	Study Objective(s)	Inclusion Criteria	Exclusion Criteria	Limitations
KSA	Mansour M. Alotaibi *et al.,*/^58^	March and April	Qualitative study	Semi-structured interviews	15 face-to-face individual interviews. / Covid-19 vaccine	To identify community pharmacists’ perceptions about providing COVID-19 vaccines in Saudi community settings.	Community pharmacists	NM	Not providing personally identifiable information for participants.
		2022							
KSA	Bander Balkhi *et al.,* /^59^	Apr-16	Cross sectional	Survey	Community pharmacists= 139/ No vaccination specification	To explore community pharmacists’ readiness to administer an immunization service in Riyadh region, Saudi Arabia	Community pharmacists across the different geographical parts of Riyadh, Saudi Arabia.	NM	NM
USA	Capurso *et al.,* /^36^	January and February 2005	Cross-sectional survey	A 15-question survey	Pharmacists	To identify the challenges to implementing a program utilizing pharmacists as immunizers.	Pharmacists from a super-market pharmacy chain in northwestern Ohio.	Others were excluded	NM
					N= 43 / No vaccination specification				
USA	Maurice N. Tran	1, 2016 to August 31, 2017	Qualitative analysis	Interviews	Pharmacists And pharmacy technicians	To assess community pharmacy staff members’ perceptions of work system factors.	Pharmacists, pharmacy technicians, and pharmacy assistants were eligible to participate.	Others were excluded	Using pharmacy specific e-mail addresses for recruitment. Geographical Limitations.
	*et al.,* /^37^				N= 7 interviews/ No vaccination specification				
USA	Tessa J. Hastings *et al.,*/^38^	NM	Randomized controlled trial	Survey	Pharmacists	To detect perceived barriers and facilitators related to immunization services before and after the We Immunize program.	Pharmacists were providing direct prescription dispensing services and offering either pneumococcal or herpes zoster vaccinations in the previous year.	Others were excluded.	Small sample size.
					N= 62 / No vaccination specification				Participants self-reported response may be subject to social desirability.
	
USA	Anne C. Pace *et al.,* /	February to March 2009	Cross sectional- Survey	Mailed survey	Pharmacists	To evaluate community pharmacists’ perceived barriers to providing immunization services.	Pharmacists who have previously taken students in the last 5 years and those that currently take students. All pharmacists who listed their practice site as a community pharmacy were identified from this list.	Others were excluded.	Moderate response rate.
	-39				N = 129/ No vaccination specification				
	


Country	Author/ citation	Date of Data collection	Study Design	Data collection form	Personnel/N / vaccination type	Study Objective(s)	Inclusion Criteria	Exclusion Criteria	Limitations
USA	Gretchen L *et al.,* /	2007	Descriptive, nonexperimental, cross-sectional study	Survey	Pharmacists	To recognize barriers to pharmacist-based immunization services in North Carolina	All pharmacists with an active pharmacist license in North Caro-Lina in early 2007.	Others were excluded	Respondents were not individually identified, which allowed for the possibility of individuals doing the survey multiple times.
	-40				N= 1274 / No vaccination specification				
USA	Salisa C. Westrick *et al.,* /^41^	April-July 2017	A mixed-mode (mail/electronic) survey	Survey	Pharmacists	To describe perceived barriers to immunization services.	They included pharmacy owners, managers, or staff pharmacists.	Others were excluded	Sampling frame error is possible.
					N= 292 / No vaccination specification				No causal relationships can be inferred.
									Pharmacy involvement in immunization services could vary from one state to another or from one location to another.
	
USA	Tessa J. Hastings *et al.,* /^42^	June to August 2014	Cross-sectional survey	Survey	Pharmacists	To assess pharmacists’ perceived barriers to providing HPV vaccination services in community pharmacies.	Pharmacy owners, managers, or staff pharmacists.	Pharmacies that did not serve the typical public (i.e., walk-in customers) or dispense medications were excluded from participation.	Pharmacists’ confidence in speaking to parents and patients about the vaccine was not addressed.
					N= 154 / HPV vaccine				A greater proportion of key informants were female.
									Social desirability.
USA	Anup Srivastav *et al.,* /^43^	February-March 2017	Cross sectional	Survey	US. clinicians (n = 1714) and pharmacists (n = 261) / No vaccination specification	To evaluate clinicians’ and pharmacists’ stated barriers to	Clinicians who provide care for adults aged 19 years in outpatient settings in general internal medicine, family medicine, Pharmacists who dispense directly to adults in outpatient settings.	NM	NM
						administering vaccination, practices, and perceptions regarding their adult patients’ attitudes toward vaccines			
USA	Benjamin S. Teeter *et al.,* /^44^	August 2019 and June 2020.	Qualitative	Interviews	Pharmacy staff, primary care clinic staff, and parents of adolescent children	To detect barriers and facilitators to providing the HPV vaccine and Vaccines for Children (VFC) program participation in pharmacies and clinics.	Pharmacy	NM	Small sample size of pharmacists, physicians, and parents from a single
					N= 29 interviews / HPV vaccine	.	managers, staff pharmacists, and pharmacy technicians.		state in the southern US.
									The risk of selection bias should be considered.
									The opinions of the those interviewed cannot be generalized to a greater population.


Country	Author/ citation	Date of Data collection	Study Design	Data collection form	Personnel/N / Vaccination type	Study Objective(s)	Inclusion Criteria	Exclusion Criteria	Limitations
USA	S. Suresh Madhavan *et al.,* /^45^	NM	Cross-sectional	Survey	Pharmacists from chain, independent, mass merchandiser/grocery, primary care clinic,	To assess the survey responses on pharmacy-based immunization services--current involvement, willingness to get involved, perceived obstacles, and patients' interest.	Randomly selected	NM	Cross-sectional survey design methodology limitations.
					and health maintenance organization settings.		nationwide sample of 5,553 pharmacists		
					N= 1348 responses / No vaccination specification				
USA	George *et al., l*^52^	November 2018 and February 2019	Cross sectional	Online survey	Pharmacists and other healthcare providers.	Measure the attitudes of pharmacists and other health care providers towards vaccine administration.	Members of Wisconsin health professional associations and immunization coalitions.	Others were excluded	Including pharmacists who are only members in PSW.
					N=236 Pharmacists				Small response number.
					N= 51 HCPs./ No vaccination specification				
USA	Philip *et al., l*^53^	July 2017 through June 2018	Cross sectional	Survey	Pharmacists	Understand the immunization barriers for Wisconsin pharmacists.	Wisconsin pharmacists	Others were excluded	Respondent pharmacies may not represent the entire state.
					N=236 / No vaccination specification				Lack of data on vaccine series completion and possible underreporting in WIR
	
USA	Jessica *et al., l*	Jun-14	Cross sectional	Semi structured survey	Pharmacists	Identify pharmacists’ challenges and facilitators perceived in-pharmacy vaccine administration.	Pharmacists previously administered or were currently administering HPV, TDAP, or MCV4 vaccines to adolescents aged 9 to 17 years.	Others were excluded	Small sample size
	-54				N=40 / No vaccination specification				
	Sandra Gerges *et al.,*^49^	2014-2015	Qualitative descriptive design	Semi structured interviews	Pharmacists	Exploring experiences and practices of pharmacist vaccinators and the effect of vaccination pain on their practice.	Pharmacists practicing in community pharmacies across the	Others were excluded	NM
Canada					N= 12 / No vaccination specification		Greater Toronto Area (GTA).		
Canada	Jennifer *et al., l*^50^	April and June 2014	Cross sectional	web-based self-administered survey	Pharmacists	Describe the experiences of pharmacists in the Canadian province of New Brunswick as immunizers	Pharmacists that are members of New Brunswick Association	Others were excluded	Insufficient survey response
					N= 168 / No vaccination specification				


Country	Author/ citation	Date of Data collection	Study Design	Data collection form	Personnel/N / Vaccination type	Study Objective(s)	Inclusion Criteria	Exclusion Criteria	Limitations
Canada	Daniyal *et al., l*^51^	Oct-20	Cross sectional	Online survey	Pharmacists	Describe the actions related to administering an injection, and to identify barriers and facilitators pharmacists face when providing injection services.	Pharmacists registered in Alberta College of pharmacy	Others were excluded	Small sample size
					N= 397 / No vaccination specification				Opinions related to injection services in general.
	
Ethiopia	Solome Tadele *et al.,* /^63^	March 25 to May 20, 2023	Online cross -sectional study	Questionnaires	Community pharmacists	Assess the barriers and willingness to implement community pharmacy-based vaccination services in Ethiopia.	Working as a community pharmacist in Ethiopia during the study period, willingness to participate in the study, and holding at least a bachelor’s degree in pharmacy	Others were excluded	Response bias.
					Total= 423 / No vaccination specification				The response rate to the survey was not reported and potential non-response bias was not discussed.
									High rate of no response.

The major study design was cross-sectional (*N* = 26) (N = 26),^8^^,^^14^^,^^16^^,^^36^^,^^38^, ^39^, ^40^^,^^42^^,^^43^^,^^45^, ^46^, ^47^, ^48^^,^^55^, ^56^, ^57^^,^^59^, ^60^, ^61^, 62., 63.^50^, ^51^, ^52^, ^53^, ^54^ Other study designs were qualitative studies (*N* = 5)^64^,^58^,^37^,^44^,^49^ mixed-methods studies (*n* = 2)^65^,^41^ and one randomized controlled trial.^38^ Most study participants were pharmacists (*n* = 29)^8^,^14^,^16^,^36^,^38^,^39^,^40^,^42^,^43^,^45^,^46^,^47^,^48^,^49^^,^^50^,^51^,^53^,^54^,^55^,^56^,^57^,^58^,^59^,^60^,^61^,^62^,^63^,^64^,^65^ except in four studies where they included “pharmacists and pharmacy technicians”, “clinicians and pharmacists”, “pharmacy staff, clinic staff, parents of adolescents children” and “and “Pharmacists and other healthcare providers” respectively^37^,^43^,^44^,^52^.

#### 3.1.1 Barriers for pharmacist-led vaccination services

This section summarizes the barriers mentioned in 32 studies out of 33 studies for pharmacist-led vaccination services as per the CFIR domains (Table 2).^19^

The FIP report identified five key barriers, three of which fall under the Outer Setting domain: limited acceptance by patients and health professionals, limited government support and limited economic support corresponding to the constructs of ‘local attitudes,’ ‘policies and laws,’ and ‘financing.’ The remaining barriers include training needs, aligning with the Inner Setting construct ‘lack of access to knowledge and information,’ and pharmacists' lack of confidence in administering vaccines, mapped to the ‘innovation deliverers’ construct.^8^

### 3.2 Innovation domain

Eight studies highlighted barriers for pharmacist administered vaccination under the Innovation Domain^36^,^41^^,^^42^,^52^, ^53^, ^54^,^59^.^61^ Three studies identified barriers mapped to the ‘complexity’ construct^59^,^61^.^54^ One study mentioned challenges associated with handling vaccines, including storage and disposal of sharp materials and with maintaining patient safety^61^ while the other study addressed compromise of patient safety^59^ and the third study highlighted the adverse reactions of vaccines.^54^ The remaining six studies stated barriers primarily related to the ‘cost’ construct. These were associated with the inability of patients to pay or to have insurance coverage for vaccination^36^,^41^^,^^42^.^52^, ^53^, ^54^

### 3.3 Outer domain

Nineteen studies identified barriers under the ‘local attitudes’ construct^8^,^39^^,^^40^,^42^,^53^^,^^54^,^44^, ^45^, ^46^, ^47^, ^48^, ^49^^,^^55^, ^56^, ^57^, ^58^,^16^^,^^61^,^65^ These were primarily related to poor collaboration or conflict with other health professionals^47^,^49^,^16^^,^^61^,^65^lack of physician support^39^^,^^40^,^45^,^48^,^55^ and limited patient demand and knowledge^42^,^44^,^46^,^53^.^54^ Ten studies had barriers linked to the ‘policy and law’ construct mainly represented by liability and regulatory issues^16^,^39^,^40^^,^^45^,^48^^,^^55^,^56^^,^^57^,^60^.^63^ Furthermore, fifteen studies stated barriers under the ‘financing construct’ mainly highlighted by inadequate reimbursement and insufficient renumeration for pharmacist vaccination services^8^,^38^,^39^,^40^,^41^,^42^,^43^,^45^,^48^,^50^,^53^,^54^,^56^,^57^.^58^

### 3.4 Inner domain

Barriers under ‘access to knowledge and information’ construct were mentioned by (*N* = 16) studies mainly in relation to the requirement for pharmacist training and the lack of time and funds for this training^8^^,^^16^,^37^,^39^,^43^,^47^,^48^,^55^, ^56^, ^57^,^59^, ^60^, ^61^, 62.^63^.^65^

Twenty-two studies also reported barriers under the ‘available resources’ construct^16^,^37^,^38^,^39^,^40^,^42^,^43^,^44^,^45^,^46^,^47^,^48^,^50^,^52^,^53^,^54^,^55^,^56^, ^57^, ^58^,^62^,^65^ particularly related to the inadequacy of pharmacy facilities and personnel resources^16^,^62^ limited space, time, staff or private areas^37^,^38^,^39^,^40^,^43^,^44^,^45^,^46^,^47^,^48^,^50^,^52^,^53^,^54^,^55^,^56^, ^57^, ^58^,^65^ unavailability of equipment for vaccine storage^43^,^44^ and vaccine expiration before use.^42^

One study identified barriers under both the ‘tension for change’ and ‘compatibility’ constructs, specifically referencing transitions backward and practicalities of giving pharmacists additional roles, respectively [51].

### 3.5 Individual characteristics

Nine studies listed barriers under ‘individual deliverers’ construct^36^,^39^,^43^,^46^,^48^,^49^,^51^,^52^.^53^ In five studies, barriers were related to capability of pharmacists to immunize^36^,^39^,^48^,^49^,^51^ while other two studies barriers were concerned with the lack of pharmacist motivation to offer vaccination^43^,^46^ and the remaining studies main barriers were related to pharmacists motivation to deliver the service^52^.^53^

#### 3.5.1 Facilitators for pharmacist-led vaccination services

This section outlines the facilitators mentioned in 16 studies supporting pharmacist-led vaccination services mapped as per the CFIR domains (Table 3).^19^

#### 3.5.2 Innovation domain

No facilitators were identified under this domain.

#### 3.5.3 Outer domain

Nine studies outlined facilitators mapped to the ‘local attitudes’ construct^14^,^16^,^49^,^58^,^59^,^60^,^61^,^62^.^65^ These included pharmacist-patient relationships or trust^49^,^58^,^65^,^60^ collaboration with other health professionals, and patient acceptance of pharmacist-administered vaccination^16^,^61^,^62^ patient demand^14^,^59^ support from health authorities, medical and nursing associations, and medical clinics^14^.^59^ Furthermore, eight studies had facilitators under the ‘financing’ construct^14^,^16^,^36^,^47^,^55^,^59^,^61^,^62^ mainly financial remuneration^16^,^47^,^59^,^62^ role of health insurance companies^55^ and comfort with the billing procedures for immunizations.^36^ And one study had facilitators mapped to policies and laws construct identified by legislative authority to provide vaccination.^54^

#### 3.5.4 Inner domain

The main facilitators were represented under the ‘access to knowledge and information’ construct in (*N* = 9) studies^14^,^16^,^36^,^38^,^47^,^55^,^59^ [47],^62^ in particular training courses for pharmacists for administering vaccinations^14^,^16^,^36^,^38^,^47^,^55^,^59^ [47].^62^Two studies discussed facilitators related to the ‘work infrastructure’ construct, mainly through offering both appointment-booking and walk-in systems,^65^ and reducing the workload of pharmacists.^61^ One study highlighted facilitators under available resources construct represented by staff time.^54^

### 3.6 Individual characteristics domain

Facilitators were mainly under the ‘innovation deliverers’ construct in eleven studies^14^,^36^,^37^,^44^,^47^,^49^,^54^,^58^,^59^,^60^.^65^ specifically under the ‘capability’, ‘motivation’ and ‘opportunity’ components, namely pharmacist specialty interest, pharmacist interest, patient trust, and demand^14^,^54^,^58^.^59^ Other facilitators were also aligned with the COM-B model including comfort with administering immunizations ‘capability’,^36^ patient-centered education ‘capability’,^37^ confidence to provide vaccines and to counsel patients ‘capability’,^44^ pharmacist personal interest ‘motivation’,^47^ and pharmacist relationship with patients and patient satisfaction ‘opportunity’.^49^

Six studies outlined facilitators under the ‘innovation recipients’ construct^16^,^51^,^58^,^59^,^60^.^61^ One facilitator was mapped to ‘opportunity’ represented by pharmacist-delivered vaccination, making it easy for patients to obtain the COVID vaccine,^60^ and two facilitators were mapped to ‘motivation’, mainly the patient acceptance of pharmacist-led vaccination^16^.^61^ Three studies had facilitators in line with ‘need’ mainly patient demand^58^,^59^ and increase in vaccination rate and lower infection rate.^51^

### 3.7 Quality of studies

There was a variation in the quality of the studies, with CCAT scores ranging between 60 % and 100 %, with one study scoring 52 %.^46^The quality scores of the studies included in this review are summarized in Table 4. The main limitations in cross-sectional studies were poor reporting of sample size, response rate, and sampling methods. Other study designs included insufficient description of participant selection and methodology.

**Table 4:** Quality of studies assessed using Crowe Critical Appraisal Tool (CCAT).

Author/Citation	Preliminaries [/5]	Introduction [/5]	Design [/5]	Sampling [/5]	Data collection [/5]	Ethical matters [/5]	Results [/5]	Discussion [/5]	Total [/40] and in %
Anne C. Pace et al.*,* /^39^	4.75	5	5	4	5	5	5	4	37.75 (94.38 %)
Adam Pattison et al.*,*^64^	4.75	5	4	5	3	5	4	5	35.75 (89.38 %)
Kevin Capurso et al.*,* /^36^	4.75	2.5	4	4	5	5	3	3	31.25 (78.13 %)
Fouad Sakr et al.*,* /^56^	5	5	4	4	5	5	5	5	38 (95.00 %)
Jean Rémi Valiquette et al.*,* /^47^	4	5	4	3	5	4	4	4	33 (82.50 %)
Gretchen et al.*,* /^40^	5	5	5	3	5	4	4	3	34 (85.00 %)
Maurice N. Tran et al.*,* /^37^	5	5	4	3	4	5	4	4	34 (85.00 %)
Tessa J. Hastings et al.*,* /^38^	4	5	5	4	5	4	5	5	37 (92.50 %)
H Laetitia Hattingh et al.*,* /^65^	5	5	5	5	5	5	5	5	40 (100.00 %)
Dalal Youssef et al.*,* /^57^	5	5	5	5	5	5	5	5	40 (100.00 %)
Nicholas Edwards et al.*,* / ^48^	5	5	5	5	5	5	5	5	40 (100.00 %)
Solome Tadele et al.*,* /^63^	4.25	5	5	5	5	5	5	5	39.75 (99.38 %)
Salisa C. Westrick et al.*,* /^41^	4.5	5	4.75	5	5	5	5	5	39.25 (98.13 %)
Benjamin S. Teeter et al.*,* / ^44^	4	5	5	5	5	5	5	5	39 (97.50 %)
S. Suresh Madhavan et al.*,* /^45^	5	5	5	5	5	5	5	5	40 (100.00 %)
Dalal Youssef et al.*,* /^14^	5	5	4.75	5	4.75	5	4	5	38.5 (96.25 %)
Nikolaus Lindner et al.*,* /^16^	3	5	2.5	3	3	5	2.5	4.75	28.75 (71.88 %)
Sandra Gerges et al.*,* /^49^	4.5	5	4.25	5	4.25	3	4	4	34 (85.00 %)
Mansour M. Alotaibi et al.*,* / ^58^	4	5	4.5	4.25	4.25	4.5	3.5	4.5	34.5 (86.25 %)
Mohammad B Nusair et al.*,* /^55^	4.5	5	4	4	4	3.5	3.5	5	33.5 (85.00 %)
Susi Ari Kristina et al.*,* /^60^	5	5	5	5	5	5	5	5	40 (100.00 %)
Tessa J. Hastings et al.*,*/^42^	5	5	5	5	4	3.5	5	5	37.5 (93.75 %)
Wei Chern Ang et al.*,* / ^61^	3	5	3	3	3	5	3.5	3.5	29 (72.50 %)
Piotr Merks et al.*,* /^62^	3	5	1.5	1.5	2.5	5	3	2.5	24 (60.00 %)
Anup Srivastav et al.*, l* ^43^	3.5	4.5	1.5	1	2.5	5	3.5	3.5	25 (62.50 %)
Bander Balkhi et al.*,* /^59^	4.5	4.5	2	1	2.5	5	2.5	2	24 (60.00 %)
Sarah E. Kelling et al.*,* /^46^	3.5	5	1.5	1	3	1.5	2	3.5	21 (52.50 %)
Helena Rosado et al.*,*/^8^	5	5	5	5	5	5	5	5	40 (100.00 %)
Jennifer et al.*, l*^50^	5	5	5	4	5	5	5	4	38 (95 %)
Daniyal et al.*, l*^51^	5	5	4	3	4	5	5	4	35 (87.5 %)
Jessica et al.*, l*^54^	5	5	4	3	5	4	5	4	35 (87.5 %)
George et al.*, l*^52^	5	5	4	3	4	5	5	4	36 (90 %)
Philip et al.*, l*^53^	5	5	4	3	4	5	5	4	35 (87.5 %)

## 4 Discussion

This systematic review synthesized global evidence on the barriers and facilitators for pharmacist-led vaccination services mapped to the CFIR framework.^19^ This review included 33 studies with diverse findings emphasizing the importance of context specific approaches to expanding pharmacist role in immunization.

According to the CFIR framework, the main barriers in the Outer Domain were mainly fear of conflicting roles with physicians and public attitudes, lack of physician support, patient unwillingness to receive vaccinations in community settings, liability and regulation related issues, and financial concerns for pharmacist vaccination services.

The external barriers under the ‘local attitudes construct’ could be primarily attributed to the lack of patient awareness about the importance of vaccination and the incapability of pharmacists to deliver such a service. The perceived incapability of pharmacists reflected public beliefs and perceptions regarding pharmacists' ability to provide the service, rather than their actual competence. Therefore, this factor was mapped to the construct of local attitudes.

This review finding is supported by a systematic review that encompassed studies from several countries, highlighting the limited appreciation and negative attitudes toward the recognition of pharmacists' roles among health professionals and the public.^66^ Similarly, a systematic review that included studies in Indonesia and Australia concluded that one of the primary challenges for pharmacist-based immunization is patients' insufficient knowledge and confidence in competencies of immunizers.^17^ Health promotion campaigns led by health ministries or pharmacy schools or pharmacist organizations to improve public awareness about pharmacist role and impact in vaccination can greatly improve local attitudes toward these services.^67^ For example, Qatar's government has initiated notable government-approved health awareness campaigns to improve adult vaccination such as those conducted by the Primary Health Care Corporation.^68^

As for regulatory issues, a recent review on pharmacist-led vaccination services equally highlighted regulatory barriers, including restrictions on pharmacists' authority to administer vaccines in several countries, such as Lebanon.^67^ Furthermore, according to the FIP report, only 11 % of surveyed countries in Europe allow pharmacists to administer vaccinations [8]. Hence, governmental support and authorization for pharmacists to administer vaccinations are essential. In fact, there are encouraging signs of small progress in this area. For instance, in the United Arab Emirates (UAE) pharmacists' roles have recently expanded to include vaccine administration. The case in the UAE may serve as an example for neighboring states demonstrating the capability of pharmacists to administer vaccinations, overcoming policy and regulatory challenges.^67^

The financial concerns identified in this review are not surprising. A recent systematic review that included studies conducted in the USA, Canada, and other countries indicated that pharmacists had difficulties obtaining reimbursement from major third-party payers.^17^ A standardized reimbursement model is essential to overcoming this challenge. A report by the FIP analyzed reimbursement models in seven countries (Australia, Canada, France, Ireland, Portugal, the UK, and the USA).^69^ In Australia vaccines under the National Immunization Program (NIP) are offered for free but pharmacists can charge administration fees if the government does not reimburse them.^69^ Canada follows a single-payer reimbursement model, meaning the insurers or government handle the process.^69^ The UK has public, private, and out-of-pocket models.^69^ The USA uses a mixed model like the UK, but coverage varies by state leading to inconsistencies in pharmacist compensation.^69^ The FIP report also mentioned key solutions to support pharmacist-led vaccination services including government authorization, standardized reimbursement rates to prevent financial penalties for pharmacies providing vaccines, and public awareness initiatives.^69^ Additionally, this report highlighted how effective reimbursement needs collaboration between government, insurers, professional organizations, and pharmacies to reduce financial barriers, enhance pharmacy services, and ultimately increase vaccination rates.^69^ Global disparities in pharmacist-led vaccination services stem from differences in regulatory authority and reimbursement structures. For instance, in countries such as the United States, Canada, and the United Kingdom, flexible regulations grant pharmacists broader authority and deeper integration into national immunization programs, contributing to higher vaccination coverage^18^.^69^ In contrast, more restrictive frameworks such as in Lebanon, where pharmacists are limited to providing immunization education and dispensing vaccines have slowed the development and implementation of such services.^56^ These discrepancies are further influenced by reimbursement policies: in the United States and Canada, pharmacists are compensated through public or insurance-based systems, while in many European and Middle Eastern countries, the absence of clear remuneration models renders these services financially unsustainable.^69^ These examples demonstrate that the successful expansion of pharmacist-led vaccination programs is closely linked to flexible legislation, supportive reimbursement structures, and strong professional representation. Therefore, adopting elements of the North American and UK models particularly in areas of regulatory advocacy and reimbursement strategies could greatly benefit countries seeking to establish or enhance such services.

In the Inner Domain, lack of pharmacist training and lack of adequate facilities for delivering community pharmacy vaccination services were the main barriers. Access to knowledge was also considered a significant barrier in a Malaysian study that demonstrated the link between the low public immunization rate and the lack of immunization education and training in schools of pharmacy across the country.^70^ Moreover, this review finding is consistent with the results of the COM-B based systematic review which reported lack of pharmacists' knowledge as a barrier for delivering vaccination services.^17^ Thus, integrating comprehensive vaccination training into pharmacy schools' curricula is vital for graduating highly skilled pharmacists capable of delivering vaccination services. Also, offering comprehensive postgraduate vaccination courses for pharmacists who wish to provide immunization services can significantly boost immunization rates and enhance pharmacists' abilities.^71^ Similarly, support from both government and private sectors in providing suitable facilities, storage, and equipment will help increase the number of community pharmacies offering pharmacist-led vaccination services.

The review findings target important critical areas for strengthening pharmacist-led vaccination services. It is anticipated that overcoming the identified barriers and nurturing the perceived facilitators for pharmacist led vaccination services would improve the public vaccination rates. For instance, a systematic review conducted in 2022 that included studies from the USA, Canada, Puerto Rico, and Japan evaluated the impact of pharmacist involvement on immunization uptake.^12^ The review concluded that pharmacists acting as immunizers, advocates, or both significantly raised immunization rates.^12^ Another systematic review, which included countries from various regions but focused mostly on North America, also highlighted the impact of pharmacists as immunizers.^72^ Interactions between domains of the CFIR framework were evident as well. For example, restrictive regulations and physician resistance (Outer Setting) might weaken pharmacists' confidence and motivation (Individual Characteristics), while lack of support from the institution as well (Inner Setting) will further constrain their readiness to activate new roles related to immunization. In contrast, the presence of Outer Setting supports, such as reimbursement policies or endorsements, strengthen Inner Setting readiness as well as increase Inner Setting (Individual Characteristics) will to provide vaccination services.

The importance of tailored strategies is underscored requiring interventions at multiple levels including legislative reform, financial incentives, interprofessional collaboration, and workforce training. Achieving this necessitates organized efforts among policymakers, pharmacy educators, and health organizations. These steps will not only support pharmacists in delivering immunizations but also encourage broader public awareness.

### 4.1 Strengths and limitations

This systematic review had several strengths. It utilized a comprehensive literature search through multiple databases and employed the updated version of the CFIR framework. This framework provided a structured and comprehensive analysis of the data, helping in identifying research gaps and in translating findings into actionable recommendations.^48^ Furthermore, the review search strategy was not limited to a specific language. As for review limitations, the major limitation was the notable lack of studies from multiple regions and countries, especially Sub-Saharan Africa, low- and middle-income countries which may reduce the external validity of the findings. Additionally, the heterogeneity of the studies and the variability in their methodological quality may have introduced potential biases, including reporting bias (from incomplete or selective reporting in studies), and publication bias (because of lack of published studies in some regions). Furthermore, we acknowledge that the lack of reporting a specific barrier or facilitator does not necessarily indicate its absence in practice and should not be interpreted as definitive evidence of nonexistence. Most included studies originated from North America, where pharmacists operate within stronger legislative frameworks and established reimbursement mechanisms. In contrast, the implementation of pharmacist-led vaccination models in Low- and Middle-Income Countries (LMICs) is hindered by weaker regulatory systems, limited infrastructure, and variable public trust in pharmacists. These contextual disparities underscore the need for regionally tailored strategies to adapt and scale pharmacist-managed vaccination frameworks effectively.

Future studies are needed to assess the barriers and facilitators for pharmacist-led vaccination services in low- and middle-income countries to assess the gaps in practice and research in these countries. More studies are also needed for designing and assessing interventions to bypass the identified barriers in this review. Examples of such studies could include implementing vaccination training programs for undergraduate pharmacy students and practicing pharmacists and evaluating their impact on vaccination rates or other related outcomes. Additional studies could be conducted to pilot pharmacist-led vaccination services and evaluate patients' perceptions of these services. Further research could also assess how community outreach initiatives aimed at educating the public about pharmacists' roles in vaccination services can influence their overall awareness. Other studies could potentially be designed to evaluate collaborative immunization models that include both pharmacists and other health professionals.

## 5 Conclusion

This study provides a comprehensive overview of the barriers and facilitators for pharmacist-led vaccination services using the CFIR framework. Barriers and facilitators were mainly under the Outer Setting Domain (local attitudes, financing, regulations and laws) and under the Inner Domain (available resources and access to knowledge). These findings present opportunities to enhance the scope and the impact of pharmacist-led immunization services by organized efforts among policymakers, pharmacy educators, and health organizations to provide targeted training, simplify reimbursement structures, and expand pharmacist authority to vaccinate. Future research should focus on addressing these barriers through targeted interventions that consider the regional specificities. Studies are also needed to assess the effectiveness of training models to improve pharmacists-led vaccination services.

## CRediT authorship contribution statement

**Meral Abdulselam:** Writing – original draft, Software, Methodology, Investigation, Formal analysis, Data curation. **Alla El-Awaisi:** Writing – review & editing, Validation, Supervision, Project administration, Methodology. **Ziad G. Nasr:** Writing – review & editing, Validation, Supervision, Project administration, Methodology. **Shahd Shaar:** Writing – review & editing, Validation, Supervision, Project administration, Methodology. **Maguy Saffouh El Hajj:** Writing – review & editing, Writing – original draft, Visualization, Validation, Supervision, Project administration, Methodology, Investigation, Formal analysis, Conceptualization.

## Funding

The open access publication of this study was possible by a student grant from Qatar University (QUST-2-CPH-2025-475).

## Declaration of competing interest

None.

## Appendix A Supplementary data

Supplementary material Image 1

Tables 1 - 2 Image 1
