# Supplementary material for: Barriers and facilitators for pharmacist-led vaccination services: A systematic review using the Consolidated Framework for Implementation Research (CFIR)
Source: Explor Res Clin Soc Pharm. 2025 Nov 25;21:100687. doi: 10.1016/j.rcsop.2025.100687 (PMC12811634; doi:10.1016/j.rcsop.2025.100687)
Supplement: Supplementary file 1 — Supplementary material [file mmc1.docx]

**appendix: SEARCH STRATEGIES**

**Supplementary: Search Terms**

**HMIC Search Terms:**

exp Vaccines/ OR exp BCG vaccines/ OR vaccine.mp.

vaccination.mp. OR exp Immunization/

influenza vaccine.mp.

hepatitis vaccine.mp.

TETANUS VACCINE.mp.

PERTUSSIS VACCINE.mp.

COVID VACCINE.mp.

PAPILLOMA VIRUS VACCINE.mp.

DENGUE VACCINE.mp.

MEASLES VACCINE.mp.

MUMPS VACCINE.mp.

RUBELLA VACCINE.mp. OR exp MMR vaccine/

VARICELLA VACCINE.mp.

HERPES VACCINE.mp.

Haemophilus influenza vaccine.mp.

pneumococcus vaccine.mp.

respiratory syncytial virus vaccine.mp.

yellow fever vaccine.mp.

polio vaccine.mp.

pharmacy.mp. OR exp Community pharmacy/ OR exp Clinical pharmacy/ OR exp Pharmacy/ OR exp Community pharmacists/ OR exp Pharmacists/ OR exp Hospital pharmacists/

exp Community pharmacy/ OR pharmacy research.mp.

(Not fully visible, but another pharmacy‐related line in the screenshot; it is combined with lines 20 and 21.)

1 OR 2 OR 3 OR 4 OR 5 OR 6 OR 7 OR 8 OR 9 OR 10 OR 11 OR 12 OR 13 OR 14 OR 15 OR 16 OR 17 OR 18 OR 19

20 OR 21 OR 22

23 AND 24

**Web of Science Search Terms:**

(AB= (Vaccine OR vaccination OR immunize OR immunization)) AND AB= (pharmacist OR pharmacy)

**DARE Search Terms:**

((vaccination OR immunization OR vaccine OR immunize):ti AND (pharmacist OR pharmacy):ti) AND ((Systematic review:ZDT AND Bibliographic:ZPS) OR (Systematic review:ZDT AND Abstract:ZPS) OR (Cochrane review:ZDT) OR (Cochrane related review record:ZDT))

**Ovid/Medline Search Terms:**

pharmacist.mp. OR exp Pharmacists

vaccine.mp. OR exp Vaccines

immunization.mp. OR exp Immunization

vaccination.mp. OR exp Vaccination

exp "Attitude of Health Personnel" OR exp Attitude OR attitude.mp.

exp Perception OR exp Social Perception

opinion.mp. OR exp Attitude

exp Pharmacy OR pharmacy.mp. OR exp Community Pharmacy Services

exp Motivation OR motivation.mp.

readiness.mp.

willingness.mp.

satisfaction.mp.

public.mp. OR exp Public Opinion

barrier.mp.

exp "Attitude of Health Personnel" OR facilitator.mp.

exp behavioral sciences/ OR exp behavioral medicine/ OR behavioral research/ OR "acceptance and commitment therapy"

exp life course perspective/ OR exp "theory of planned behavior"/

exp Awareness/ OR awareness.mp.

understanding.mp. OR exp Comprehension/

feeling.mp. OR exp Emotions/

recognition.mp. OR exp Recognition, Psychology/

observation.mp. OR exp Behavior Observation Techniques/ OR exp Observation/

insight.mp.

Health Knowledge, Attitudes, Practice/

impression.mp.

1 OR 8

2 OR 3 OR 4

challenge.mp.

5 OR 6 OR 7 OR 9 OR 10 OR 11 OR 12 OR 13 OR 14 OR 15 OR 16 OR 17 OR 18 OR 19 OR 20 OR 21 OR 22 OR 23 OR 24 OR 25 OR 28

26 AND 27 AND 29

**Embase Search Terms:**

#1 ‘vaccination’/exp OR ‘vaccination’

#2 ‘vaccine’

#3 ‘immunization’

#4 immunize

#5 ‘pharmacist’

#6 pharmacy

#7 community AND pharmacy AND services

#8 ‘pharmacy research’

#9 ‘hospital pharmacy’

#10 ‘influenza vaccine’

#11 ‘hpv vaccination’

#12 ‘hepatitis vaccine’

#13 ‘human immunodeficiency virus vaccine’

#14 ‘measles vaccine’

#15 ‘mumps vaccine’

#16 ‘rubella vaccine’

#17 ‘meningococcus vaccine’

#18 ‘monkeypox vaccine’

#19 ‘poliomyelitis vaccine’

#20 ‘pneumococcus vaccine’

#21 ‘pertussis vaccine’

#22 ‘tetanus toxoid’

#23 chickenpox AND vaccin

#24 ‘varicella zoster vaccine’

#25 ‘haemophilus vaccine’

#26 ‘rotavirus vaccine’

#27 ‘dengue vaccine’

#28 *(Combines vaccination-related terms)*

#1 OR #2 OR #3 OR #4

OR #10 OR #11 OR #12 OR #13 OR #14 OR #15

OR #16 OR #17 OR #18 OR #19 OR #20 OR #21

OR #22 OR #23 OR #24 OR #25 OR #26 OR #27

#29
*(Combines pharmacy-related terms)*

#5 OR #6 OR #7 OR #8 OR #9

#30
*(Intersection of vaccination and pharmacy sets)*

#28 AND #29

#31
*(Limits to article types)*

#30 AND

(‘article’/it OR ‘article in press’/it

OR ‘conference abstract’/it OR ‘conference paper’/it

OR ‘conference review’/it OR ‘note’/it

OR ‘preprint’/it OR ‘short survey’/it).

**Google Scholar search terms:**

Vaccine and Pharmacist

**Cochrane Library:**

#1 (“vaccine*”) OR (vaccination) OR (immunization) OR (immunize)

#2 pharmacist OR pharmacy OR community pharmacy services OR hospital pharmacy services

OR pharmacy research

#3 #1 AND #2

**CINAHL Search Terms:**

S1 (vaccines/immunization terms):

vaccine OR vaccination OR immunization OR immunize

OR influenza vaccine OR hpv vaccine OR hiv vaccine

OR hepatitis vaccine OR measles vaccine OR mumps vaccine

OR rubella vaccine OR meningococcal vaccine

S2 (pharmacy terms):

pharmacist OR pharmacy OR community pharmacy service OR hospital pharmacy service

OR pharmacy research OR pharmacy services

S3 (combined search):

S1 AND S2

**Prospero Search Term:**

(Vaccination OR Vaccine OR immunization OR immunize*) AND (pharmacist OR pharmacy)
