# Supplementary material for: Barriers and facilitators for pharmacist-led vaccination services: A systematic review using the Consolidated Framework for Implementation Research (CFIR)
Source: Explor Res Clin Soc Pharm. 2025 Nov 25;21:100687. doi: 10.1016/j.rcsop.2025.100687 (PMC12811634; doi:10.1016/j.rcsop.2025.100687)
Supplement: Supplementary file 2 — Tables 1 - 2 [file mmc2.docx]

| **Table 2. Barriers for Pharmacist-led Vaccination Services** | | **I. INNOVATION DOMAIN** | | | | | | | | | | | | **II. OUTER SETTING DOMAIN** | | | | | | | | | | **III. INNER SETTING DOMAIN** | | | | | | | | | | | | | | | | **IV.**  **INDIVIDUAL DOMAIN** | | |
| --- | --- | --- | --- | --- | --- | --- | --- | --- | --- | --- | --- | --- | --- | --- | --- | --- | --- | --- | --- | --- | --- | --- | --- | --- | --- | --- | --- | --- | --- | --- | --- | --- | --- | --- | --- | --- | --- | --- | --- | --- | --- | --- |
| **Author/**  **Country/**  **Citation** | **Study reported Barriers** | **A. Source** | | **B. Evidence-Base** | | **C. Relative Advantage** | | **D. Adaptability** | | **E. Trialability** | **F. Complexity** | **G. Design** | **H. Cost** | **A. Critical Incidents** | **B. Local Attitudes** | **C. Local Conditions** | **D. Partnership & Connections** | **E. Policies & Laws** | **F. Financing** | **External pressure** | **G1. Societal Pressure** | **G2. Market Pressure** | **G3. Performance-Measurement Pressure** | **1. Physical Infrastructure** | **2.Information Technology Infrastructure** | **3. Work Infrastructure** | **B. Relational Connections** | **C. Communications** | **D. Culture** | **E. Tension for Change** | **F. Compatibility** | **G. Relative Priority** | | **H. Incentive Systems** | | **I. Mission Alignment** | | **J. Available resources** | **K. Access to Knowledge & Information** | **A. High-level Leaders*** | **H. Innovation Recipients** | **I. Innovation deliverers** |
| Helena Rosado *et al.,* / Global  (8) | Limited acceptance by government |  | | | | | | | | |  |  |  |  |  |  |  | **^X^** |  |  |  |  |  |  |  |  |  |  |  |  |  | | | | | | |  |  |  |  |  |
|  | Limited acceptance by health professionals and patients |  |  |  |  |  |  |  |  |  |  |  |  |  | **^X^** |  |  |  |  |  |  |  |  |  |  |  |  |  |  |  |  | | | | | | |  |  |  |  |  |
|  | Limited support economically |  |  |  |  |  |  |  |  |  |  |  |  |  |  |  |  |  | **^X^** |  |  |  |  |  |  |  |  |  |  |  |  | | | | | | |  |  |  |  |  |
|  | Limited due to training needs |  |  |  |  |  |  |  |  |  |  |  |  |  |  |  |  |  |  |  |  |  |  |  |  |  |  |  |  |  |  | | | | | | |  | **^X^** |  |  |  |
|  | Limited due to lack of confidence by pharmacist |  |  |  |  |  |  |  |  |  |  |  |  |  |  |  |  |  |  |  |  |  |  |  |  |  |  |  |  |  |  | | | | | | |  |  |  |  | **^C^** |
| H Laetitia Hattingh *et al.,* /  Australia (65) | Unavailability of stock |  | | | | | | | | |  |  |  |  |  |  |  |  |  |  |  |  |  |  |  |  |  |  |  |  |  | | | | | | | **^X^** |  |  |  |  |
|  | Fear of jeopardizing relationships with GPs |  |  |  |  |  |  |  |  |  |  |  |  |  | **^X^** |  |  |  |  |  |  |  |  |  |  |  |  |  |  |  |  |  |  |  |  |  |  |  |  |  |  |  |
|  | Competitive pricing |  |  |  |  |  |  |  |  |  |  |  |  |  |  |  |  |  |  |  |  | **^x^** |  |  |  |  |  |  |  |  |  |  |  |  |  |  |  |  |  |  |  |  |
|  | Training fees |  |  |  |  |  |  |  |  |  |  |  |  |  |  |  |  |  |  |  |  |  |  |  |  |  |  |  |  |  |  |  |  |  |  |  |  |  | **^x^** |  |  |  |
|  | Workforce issues |  |  |  |  |  |  |  |  |  |  |  |  |  |  |  |  |  |  |  |  |  |  |  |  | **^x^** |  |  |  |  |  |  |  |  |  |  |  |  |  |  |  |  |
|  | Pharmacy physical layout issues |  |  |  |  |  |  |  |  |  |  |  |  |  |  |  |  |  |  |  |  |  |  | **^X^** |  |  |  |  |  |  |  |  |  |  |  |  |  |  |  |  |  |  |
| Susi Ari Kristina *et al.,* /  Indonesia (60) | Lack of training and skills in administering vaccines (3.84±1.13) |  | | | | | | | | |  |  |  |  |  |  |  |  |  |  |  |  |  |  |  |  |  |  |  |  |  | | | | | | |  | **^x^** |  |  |  |
|  | Lack of confidence in administering vaccinations directly to patients (3.64±1.04) |  |  |  |  |  |  |  |  |  |  |  |  |  |  |  |  |  |  |  |  |  |  |  |  |  |  |  |  |  |  |  |  |  |  |  |  |  |  |  |  | **^C^** |
|  | Regulation for pharmacists to provide vaccination (3.74±1.11) |  |  |  |  |  |  |  |  |  |  |  |  |  |  |  |  | **^x^** |  |  |  |  |  |  |  |  |  |  |  |  |  |  |  |  |  |  |  |  |  |  |  |  |
|  | Collaboration with other health workers (3.56±1.09) |  |  |  |  |  |  |  |  |  |  |  |  |  | **^X^** |  |  |  |  |  |  |  |  |  |  |  |  |  |  |  |  |  |  |  |  |  |  |  |  |  |  |  |
| Wei Chern Ang *et al., /*  Malaysia (61) | Lack of pharmacists training (52.8% strongly agreed; 40.4% agreed) |  | | | | | | | | |  |  |  |  |  |  |  |  |  |  |  |  |  |  |  |  |  |  |  |  |  | | | | | | |  | **^x^** |  |  |  |
|  | Handling vaccines, storage, and disposal of sharp items (25.8% strongly agreeing and 40.0% agreeing) |  |  |  |  |  |  |  |  |  | ^x^ |  |  |  |  |  |  |  |  |  |  |  |  |  |  |  |  |  |  |  |  |  |  |  |  |  |  |  |  |  |  |  |
|  | Conflict with other health professionals who are eligible to vaccinate (6.5% strongly agreeing and 35.6% agreeing) |  |  |  |  |  |  |  |  |  |  |  |  |  | **^x^** |  |  |  |  |  |  |  |  |  |  |  |  |  |  |  |  |  |  |  |  |  |  |  |  |  |  |  |
|  | Patient safety might be compromised (7.8% strongly agreed; 45.9% agreed) |  |  |  |  |  |  |  |  |  | ^X^ |  |  |  |  |  |  |  |  |  |  |  |  |  |  |  |  |  |  |  |  |  |  |  |  |  |  |  |  |  |  |  |
| **Table 2. Barriers for Pharmacist-led Vaccination Services** | | **I. INNOVATION DOMAIN** | | | | | | | | | | | | **II. OUTER SETTING DOMAIN** | | | | | | | | | | **III. INNER SETTING DOMAIN** | | | | | | | | | | | | | | | | **IV.**  **INDIVIDUAL DOMAIN** | | |
| **Author/**  **Country**  **/Citation** | **Study reported Barriers** | **A. Source** | **B. Evidence-Base** | | **C. Relative Advantage** | | **D. Adaptability** | | **E. Trialability** | | **F. Complexity** | **G. Design** | **H. Cost** | **A. Critical Incidents** | **B. Local Attitudes** | **C. Local Conditions** | **D. Partnership & Connections** | **E. Policies & Laws** | **F. Financing** | **External pressure** | **G1. Societal Pressure** | **G2. Market Pressure** | **G3. Performance-Measurement Pressure** | **1. Physical Infrastructure** | **2.Information Technology Infrastructure** | **3. Work Infrastructure** | **B. Relational Connections** | **C. Communications** | **D. Culture** | **E. Tension for Change** | **F. Compatibility** | | **G. Relative Priority** | | **H. Incentive Systems** | | **I. Mission Alignment** | **J. Available resources** | **K. Access to Knowledge & Information** | **A. High-level Leaders*** | **H. Innovation Recipients** | **I. Innovation deliverers** |
| Nikolaus Lindner *et al.* /Austria (16) | Legal liability (54.7%) |  | | | | | | | | |  |  |  |  |  |  |  | **^x^** |  |  |  | | |  |  |  |  |  |  |  |  | | | | | | |  |  |  |  |  |
|  | Conflict with other health professionals (68.4%) |  |  |  |  |  |  |  |  |  |  |  |  |  |  | **^x^** |  |  |  |  |  |  |  |  |  |  |  |  |  |  |  |  |  |  |  |  |  |  |  |  |  |  |
|  | Management of side effects (64.8%) |  |  |  |  |  |  |  |  |  |  |  |  |  |  |  |  |  |  |  |  |  |  |  |  |  |  |  |  |  |  |  |  |  |  |  |  |  |  |  |  | **^C^** |
|  | Adequate pharmacy premises (61.6%) and Personnel resources (61.6%) |  |  |  |  |  |  |  |  |  |  |  |  |  |  |  |  |  |  |  |  |  |  |  |  |  |  |  |  |  |  |  |  |  |  |  |  | **^X^** |  |  |  |  |
|  | Appropriate training (56.4%) |  |  |  |  |  |  |  |  |  |  |  |  |  |  |  |  |  |  |  |  |  |  |  |  |  |  |  |  |  |  |  |  |  |  |  |  |  | **^x^** |  |  |  |
| Piotr Merks *et al.,* /  Poland(62) | Increased Workload (67.5%) |  | | | | | | | | |  |  |  |  |  |  |  |  |  |  |  | | |  |  | **^X^** |  |  |  |  |  | | | | | | |  |  |  |  |  |
|  | Insufficient Training Courses (66.5%) |  |  |  |  |  |  |  |  |  |  |  |  |  |  |  |  |  |  |  |  |  |  |  |  |  |  |  |  |  |  |  |  |  |  |  |  |  | **^x^** |  |  |  |
|  | Inadequate Pharmacy Facilities (70.3 %) |  |  |  |  |  |  |  |  |  |  |  |  |  |  |  |  |  |  |  |  |  |  | **^x^** |  |  |  |  |  |  |  |  |  |  |  |  |  | **^x^** |  |  |  |  |
| Adam Pattison *et al.,* /  UK (64) | Practicalities of additional roles |  | | | | | | | | |  |  |  |  |  |  |  |  |  |  |  | | |  |  |  |  |  |  |  | **^x^** |  | |  | |  | |  |  |  |  |  |
|  | Transitions backward |  |  |  |  |  |  |  |  |  |  |  |  |  |  |  |  |  |  |  |  |  |  |  |  |  |  |  |  | **^X^** |  |  |  |  |  |  |  |  |  |  |  |  |
| Mohammad B Nusair *et al.,* /  Jordan (55) | Physicians would object the idea of pharmacists becoming immunizers. |  | | | | | | | | |  |  |  |  | **^x^** |  |  |  |  |  |  | | |  |  |  |  |  |  |  |  | | | | | | |  |  |  |  |  |
|  | Insufficient experience |  |  |  |  |  |  |  |  |  |  |  |  |  |  |  |  |  |  |  |  |  |  |  |  |  |  |  |  |  |  |  |  |  |  |  |  |  | **^x^** |  |  | **^C^** |
|  | Lack of private areas |  |  |  |  |  |  |  |  |  |  |  |  |  |  |  |  |  |  |  |  |  |  |  |  |  |  |  |  |  |  |  |  |  |  |  |  | **^X^** |  |  |  |  |
|  | Regulations and liability |  |  |  |  |  |  |  |  |  |  |  |  |  |  |  |  | **^X^** |  |  |  |  |  |  |  |  |  |  |  |  |  |  |  |  |  |  |  |  |  |  |  |  |
| Fouad Sakr *et al.,* /  Lebanon (56) | Lack of support from physicians (57.1%). And Lack of support from management (22.2%) |  | | | | | | | | |  |  |  |  | ^x^ |  |  |  |  |  |  | | |  |  |  |  |  |  |  |  | | | | | | |  |  |  |  |  |
|  | lack of Space (25.4%.) |  |  |  |  |  |  |  |  |  |  |  |  |  |  |  |  |  |  |  |  |  |  |  |  |  |  |  |  |  |  |  |  |  |  |  |  | ^x^ |  |  |  |  |
|  | Time needed for professional development and training (34.9%) and Cost associated with professional development and additional training (21.3%). |  |  |  |  |  |  |  |  |  |  |  |  |  |  |  |  |  |  |  |  |  |  |  |  |  |  |  |  |  |  |  |  |  |  |  |  |  | ^x^ |  |  |  |
|  | Reimbursement concerns (23.8%) |  |  |  |  |  |  |  |  |  |  |  |  |  |  |  |  |  | ^x^ |  |  |  |  |  |  |  |  |  |  |  |  |  |  |  |  |  |  |  |  |  |  |  |
|  | Liability and malpractice concerns (20.3%) |  |  |  |  |  |  |  |  |  |  |  |  |  |  |  |  | ^x^ |  |  |  |  |  |  |  |  |  |  |  |  |  |  |  |  |  |  |  |  |  |  |  |  |
| **Table 2. Barriers for Pharmacist-led Vaccination Services** | | **I. INNOVATION DOMAIN** | | | | | | | | | | | | **II. OUTER SETTING DOMAIN** | | | | | | | | | | **III. INNER SETTING DOMAIN** | | | | | | | | | | | | | | | | **IV.**  **INDIVIDUAL DOMAIN** | | |
| **Author/**  **Country/**  **Citation** | **Study reported Barriers** | **A. Source** | **B. Evidence-Base** | | **C. Relative Advantage** | | **D. Adaptability** | | **E. Trialability** | | **F. Complexity** | **G. Design** | **H. Cost** | **A. Critical Incidents** | **B. Local Attitudes** | **C. Local Conditions** | **D. Partnership & Connections** | **E. Policies & Laws** | **F. Financing** | **External pressure** | **G1. Societal Pressure** | **G2. Market Pressure** | **G3. Performance-Measurement Pressure** | **1. Physical Infrastructure** | **2.Information Technology Infrastructure** | **3. Work Infrastructure** | **B. Relational Connections** | **C. Communications** | **D. Culture** | **E. Tension for Change** | **F. Compatibility** | | **G. Relative Priority** | | **H. Incentive Systems** | | **I. Mission Alignment** | **J. Available resources** | **K. Access to Knowledge & Information** | **A. High-level Leaders*** | **H. Innovation Recipients** | **I. Innovation deliverers** |
| Dalal Youssef *et al.,* /  Lebanon (57) | Conflicts with other professionals (75%). |  | | | | | | | | |  |  |  |  | ^x^ |  |  |  |  |  |  | | |  |  |  |  |  |  |  |  | | | | | | |  |  |  |  |  |
|  | Liability and malpractice concerns (54.6%), |  |  |  |  |  |  |  |  |  |  |  |  |  |  |  |  | ^x^ |  |  |  | | |  |  |  |  |  |  |  |  |  |  |  |  |  |  |  |  |  |  |  |
|  | Time and cost needed for professional development and training (44.4%) |  |  |  |  |  |  |  |  |  |  |  |  |  |  |  |  |  |  |  |  | | |  |  |  |  |  |  |  |  |  |  |  |  |  |  |  | ^x^ |  |  |  |
|  | Insufficient human resources (20.9%)  And lack of space (11.9%) |  |  |  |  |  |  |  |  |  |  |  |  |  |  |  |  |  |  |  |  | | |  |  |  |  |  |  |  |  |  |  |  |  |  |  | ^x^ |  |  |  |  |
|  | Remuneration (49%) |  |  |  |  |  |  |  |  |  |  |  |  |  |  |  |  |  | ^x^ |  |  | | |  |  |  |  |  |  |  |  |  |  |  |  |  |  |  |  |  |  |  |
|  | Lack of knowledge of how to manage adverse events after immunizing (41.3%) |  |  |  |  |  |  |  |  |  |  |  |  |  |  |  |  |  |  |  |  | | |  |  |  |  |  |  |  |  |  |  |  |  |  |  |  |  |  |  | ^C^ |
| Mansour M. Alotaibi *et al.,* / Kingdom of Saudi Arabia (58) | Workload and capacity |  | | | | | | | | |  |  |  |  |  |  |  |  |  |  |  | | |  |  | ^x^ |  |  |  |  |  | | | | | | |  |  |  |  |  |
|  | Availability of an adequate space in which to provide the service, and storage. |  |  |  |  |  |  |  |  |  |  |  |  |  |  |  |  |  |  |  |  |  |  |  |  |  |  |  |  |  |  |  |  |  |  |  |  | ^x^ |  |  |  |  |
|  | Some participants indicated that people might not be willing to get vaccines in community pharmacies, as they were not nationally well known for providing such a service. |  |  |  |  |  |  |  |  |  |  |  |  |  | ^x^ |  |  |  |  |  |  |  |  |  |  |  |  |  |  |  |  |  |  |  |  |  |  |  |  |  |  |  |
|  | Service was not adequately rewarded. |  |  |  |  |  |  |  |  |  |  |  |  |  |  |  |  |  | ^xx^ |  |  |  |  |  |  |  |  |  |  |  |  |  |  |  |  |  |  |  |  |  |  |  |
|  | Competency of community pharmacists to provide the service |  |  |  |  |  |  |  |  |  |  |  |  |  |  |  |  |  |  |  |  |  |  |  |  |  |  |  |  |  |  |  |  |  |  |  |  |  |  |  |  | ^C^ |
| Bander Balkhi  *et al.,* **/** Kingdom of  Saudi Arabia (59) | Lack of training to deliver vaccination services (75.4%) |  | | | | | | | | |  |  |  |  |  |  |  |  |  |  |  | | |  |  |  |  |  |  |  |  | | | | | | |  | ^x^ |  |  |  |
|  | Patient safety may be compromised (67.4%) |  |  |  |  |  |  |  |  |  | ^x^ |  |  |  |  |  |  |  |  |  |  |  |  |  |  |  |  |  |  |  |  |  |  |  |  |  |  |  |  |  |  |  |
| Kevin A. Capurso *et al.,* /  USA (36) | Cost was the main determining factor whether patients would receive a vaccine from a pharmacist (48.8%) |  | | | | | | | | |  |  | ^x^ |  |  |  |  |  |  |  |  | | |  |  |  |  |  |  |  |  | | | | | | |  |  |  |  |  |
|  | Pharmacists are uncomfortable with administering immunization (41.9%) and Pharmacists were concerned about the risk of adverse reactions to the vaccines (79.1%). |  |  |  |  |  |  |  |  |  |  |  |  |  |  |  |  |  |  |  |  |  |  |  |  |  |  |  |  |  |  |  |  |  |  |  |  |  |  |  |  | ^c^ |
|  | Patient privacy was an issue (65.1%). |  |  |  |  |  |  |  |  |  |  |  |  |  |  |  |  |  |  |  |  |  |  |  |  |  |  |  | ^x^ |  |  |  |  |  |  |  |  |  |  |  |  |  |
| Maurice N. Tran  *et al.,* /  USA (37) | SEIPS 2.0 domain: Tasks (Lack of time was acknowledged to be the biggest challenge to the consistent provision of patient-centered education. |  | | | | | | | | |  |  |  |  |  |  |  |  |  |  |  | | |  |  |  |  |  |  |  |  | | | | | | | ^x^ |  |  |  |  |
|  | SEIPS 2.0 domain: Organization  Staff training: scheduling of staff training. |  |  |  |  |  |  |  |  |  |  |  |  |  |  |  |  |  |  |  |  |  |  |  |  |  |  |  |  |  |  |  |  |  |  |  |  |  | ^x^ |  |  |  |
| Jennifer *et al., l* Canada  (50) | Reimbursement concerns (66%) |  | | | | | | | | |  |  |  |  |  |  |  |  | ^x^ |  |  | | |  |  |  |  |  |  |  |  | | | | | | |  |  |  |  |  |
|  | Lack of universal influenza vaccination (44%)  Insufficient staffing (42%) |  |  |  |  |  |  |  |  |  |  |  |  |  |  |  |  |  |  |  |  |  |  |  |  |  |  |  |  |  |  |  |  |  |  |  |  | ^x^ |  |  |  |  |
| Daniyal *et al., l* Canada (51) | Comfort with administering injections (p<0.001)  Managing adverse events (P<0.048) |  | | | | | | | | |  |  |  |  |  |  |  |  |  |  |  | | |  |  |  |  |  |  |  |  | | | | | | |  |  |  |  | ^c^ |
| Jessica *et al., l* USA  (54) | Patient afraid adult (15%), adolescent (53%) and Education of patient adult (40%), adolescent (30%). |  | | | | | | | | |  |  |  |  | ^x^ |  |  |  |  |  |  | | |  |  |  |  |  |  |  |  | | | | | | |  |  |  |  |  |
|  | Reimbursement adult (78%), adolescent (28%). |  |  |  |  |  |  |  |  |  |  |  |  |  |  |  |  |  | ^x^ |  |  |  |  |  |  |  |  |  |  |  |  |  |  |  |  |  |  |  |  |  |  |  |
|  | Time constraints of pharmacists, adult (35%), adolescent (28%).  Availability of administration area/vaccine shortage/cost of vaccine storage adult (43%), adolescent (5%). |  |  |  |  |  |  |  |  |  |  |  |  |  |  |  |  |  |  |  |  |  |  |  |  |  |  |  |  |  |  |  |  |  |  |  |  | ^x^ |  |  |  |  |
|  | Cost to patient adult (25%), adolescent (15%). |  |  |  |  |  |  |  |  |  |  |  | ^x^ |  |  |  |  |  |  |  |  |  |  |  |  |  |  |  |  |  |  |  |  |  |  |  |  |  |  |  |  |  |
|  | Adverse drug reactions, adult (20%), adolescent (20%). |  |  |  |  |  |  |  |  |  | ^x^ |  |  |  |  |  |  |  |  |  |  |  |  |  |  |  |  |  |  |  |  |  |  |  |  |  |  |  |  |  |  |  |
| George *et al., l* USA (52) | Patient refusal due to financial reasons (55%)  Patients having insurance coverage for vaccines (55%) |  | | | | | | | | |  |  | ^x^ |  |  |  |  |  |  |  |  | | |  |  |  |  |  |  |  |  | | | | | | |  |  |  |  |  |
|  | Having enough staff to provide vaccines |  |  |  |  |  |  |  |  |  |  |  |  |  |  |  |  |  |  |  |  |  |  |  |  |  |  |  |  |  |  |  |  |  |  |  |  | ^x^ |  |  |  |  |
|  | Other responsibilities taking precedence over vaccinating (52%) |  |  |  |  |  |  |  |  |  |  |  |  |  |  |  |  |  |  |  |  |  |  |  |  |  |  |  |  |  |  |  |  |  |  |  |  |  |  |  |  | ^x^ |
| Philip *et al., l* USA (53) | Patients having insurance coverage for vaccines (90%).  Patients refusing vaccines for financial reasons (89%). |  | | | | | | | | |  |  | ^x^ |  |  |  |  |  |  |  |  | | |  |  |  |  |  |  |  |  | | | | | | |  |  |  |  |  |
|  | Patients refusing vaccines (89%).  Patients refusing vaccines due to perceived safety issues (79%). |  |  |  |  |  |  |  |  |  |  |  |  |  | ^x^ |  |  |  |  |  |  |  |  |  |  |  |  |  |  |  |  |  |  |  |  |  |  |  |  |  |  |  |
|  | Having enough staff to provide vaccines |  |  |  |  |  |  |  |  |  |  |  |  |  |  |  |  |  |  |  |  |  |  |  |  |  |  |  |  |  |  |  |  |  |  |  |  |  |  |  | ^M^ |  |
|  | Other responsibilities taking precedence over vaccinating (84%) |  |  |  |  |  |  |  |  |  |  |  |  |  |  |  |  |  |  |  |  |  |  |  |  |  |  |  |  |  |  |  |  |  |  |  |  |  |  |  |  |  |
|  | Reimbursement by insurance companies (87%). |  |  |  |  |  |  |  |  |  |  |  |  |  |  |  |  | ^x^ |  |  |  |  |  |  |  |  |  |  |  |  |  |  |  |  |  |  |  |  |  |  |  | ^N^ |
| **Table 2. Barriers for Pharmacist-led Vaccination Services** | | **I. INNOVATION DOMAIN** | | | | | | | | | | | | **II. OUTER SETTING DOMAIN** | | | | | | | | | | **III. INNER SETTING DOMAIN** | | | | | | | | | | | | | | | | **IV.**  **INDIVIDUAL DOMAIN** | | |
| **Author/Country/Citation** | **Study reported Barriers** | **A. Source** | **B. Evidence-Base** | | **C. Relative Advantage** | | **D. Adaptability** | | **E. Trialability** | | **F. Complexity** | **G. Design** | **H. Cost** | **A. Critical Incidents** | **B. Local Attitudes** | **C. Local Conditions** | **D. Partnership & Connections** | **E. Policies & Laws** | **F. Financing** | **External pressure** | **G1. Societal Pressure** | **G2. Market Pressure** | **G3. Performance-Measurement Pressure** | **1. Physical Infrastructure** | **2.Information Technology Infrastructure** | **3. Work Infrastructure** | **B. Relational Connections** | **C. Communications** | **D. Culture** | **E. Tension for Change** | **F. Compatibility** | | **G. Relative Priority** | | **H. Incentive Systems** | | **I. Mission Alignment** | **J. Available resources** | **K. Access to Knowledge & Information** | **A. High-level Leaders*** | **H. Innovation Recipients** | **I. Innovation deliverers** |
| Anne C. Pace *et al.,* /  USA (39) | Staff support: Chain/grocery store (47%/55%), Independent pharmacy/mass merchandiser (29%/25%).  Physician support: Grocery chain (24%), Chain (24%), |  | | | | | | | | |  |  |  |  | ^x^ |  |  |  |  |  |  | | |  |  |  |  |  |  |  |  | | | | | | |  |  |  |  |  |
|  | Time: Chain (89%), grocery store (73%), independent pharmacy/mass merchandiser (29%/25%)  Space: Grocery chain (45%), Chain (47%), Independent pharmacy (26%) and Mass merchandiser (25%). |  |  |  |  |  |  |  |  |  |  |  |  |  |  |  |  |  |  |  |  |  |  |  |  |  |  |  |  |  |  |  |  |  |  |  |  | ^x^ |  |  |  |  |
|  | Level of knowledge/training: Grocery chain (29%), Chain (45%) and independent pharmacy (29%). |  |  |  |  |  |  |  |  |  |  |  |  |  |  |  |  |  |  |  |  |  |  |  |  |  |  |  |  |  |  |  |  |  |  |  |  |  | ^x^ |  |  | ^C^ |
|  | Legal Liability: Grocery chain (9%), Chain (50%), Independent pharmacy (41%) and Mass merchandiser (50%). |  |  |  |  |  |  |  |  |  |  |  |  |  |  |  |  | ^x^ |  |  |  |  |  |  |  |  |  |  |  |  |  |  |  |  |  |  |  |  |  |  |  |  |
|  | Reimbursement: Grocery chain (36%), Chain (32%), Independent pharmacy (61%) and Mass merchandiser (25%). |  |  |  |  |  |  |  |  |  |  |  |  |  |  |  |  |  | ^x^ |  |  |  |  |  |  |  |  |  |  |  |  |  |  |  |  |  |  |  |  |  |  |  |
| Gretchen L Kummer *et al.,* /  USA (40) | Active immunizers: Time 52.6% and area/space 25.2%.  Inactive immunizers: Time (54.3%) and space (39%)  Active immunizers: Availability of vaccine (43%) |  | | | | | | | | |  |  |  |  |  |  |  |  |  |  |  | | |  |  |  |  |  |  |  |  | | | | | | | ^x^ |  |  |  |  |
|  | Active immunizers: Availability of physician support (24.4%)  Inactive immunizers: Staff support (36.2%), Owner or Manager support (33.3%) |  |  |  |  |  |  |  |  |  |  |  |  |  | ^x^ |  |  |  |  |  |  |  |  |  |  |  |  |  |  |  |  |  |  |  |  |  |  |  |  |  |  |  |
|  | Active immunizers: Inability to obtain reimbursement from major third-party payers (68.2%)  Inactive immunizers: Level of reimbursement (32.4%) |  |  |  |  |  |  |  |  |  |  |  |  |  |  |  |  |  | ^x^ |  |  |  |  |  |  |  |  |  |  |  |  |  |  |  |  |  |  |  |  |  |  |  |
|  | Active immunizers: Pharmacists are prohibited from administering vaccines to patients under 18 years of age (59.9%) |  |  |  |  |  |  |  |  |  |  |  |  |  |  |  |  | ^x^ |  |  |  |  |  |  |  |  |  |  |  |  |  |  |  |  |  |  |  |  |  |  |  |  |
|  |  |  |  |  |  |  |  |  |  |  |  |  |  |  |  |  |  |  |  |  |  |  |  |  |  |  |  |  |  |  |  |  |  |  |  |  |  |  |  |  |  |  |
| Salisa C. Westrick *et al.,* / A33  USA (41) | Patient-related factors: ‘‘patients not having insurance coverage for vaccines (22.3%)” and ‘‘patient refusing vaccines for financial reasons (16.8%)” |  | | | | | | | | |  |  | ^x^ |  |  |  |  |  |  |  |  | | |  |  |  |  |  |  |  |  | | | | | | |  |  |  |  |  |
|  | Organizational and environmental factors: The potential financial loss due to vaccine expiration (38.7%), lack of adequate reimbursement for vaccine administration (26.4%), upfront cost of buying vaccines (26.4%). |  |  |  |  |  |  |  |  |  |  |  |  |  |  |  |  |  | ^x^ |  |  |  |  |  |  |  |  |  |  |  |  |  |  |  |  |  |  |  |  |  |  |  |
| **Table 2. Barriers for Pharmacist-led Vaccination Services** | | **I. INNOVATION DOMAIN** | | | | | | | | | | | | **II. OUTER SETTING DOMAIN** | | | | | | | | | | **III. INNER SETTING DOMAIN** | | | | | | | | | | | | | | | | **IV.**  **INDIVIDUAL DOMAIN** | | |
| **Author/Country/Citation** | **Study reported Barriers** | **A. Source** | **B. Evidence-Base** | | **C. Relative Advantage** | | **D. Adaptability** | | **E. Trialability** | | **F. Complexity** | **G. Design** | **H. Cost** | **A. Critical Incidents** | **B. Local Attitudes** | **C. Local Conditions** | **D. Partnership & Connections** | **E. Policies & Laws** | **F. Financing** | **External pressure** | **G1. Societal Pressure** | **G2. Market Pressure** | **G3. Performance-Measurement Pressure** | **1. Physical Infrastructure** | **2.Information Technology Infrastructure** | **3. Work Infrastructure** | **B. Relational Connections** | **C. Communications** | **D. Culture** | **E. Tension for Change** | **F. Compatibility** | | **G. Relative Priority** | | **H. Incentive Systems** | | **I. Mission Alignment** | **J. Available resources** | **K. Access to Knowledge & Information** | **A. High-level Leaders*** | **H. Innovation Recipients** | **I. Innovation deliverers** |
| Tessa J. Hastings *et al.,* /  USA (42) | Lack of patients who want the HPV vaccine (56.5%), Parental concerns about safety (78.7%),  Lack adequate education about the HPV infection (86.6%). |  | | | | | | | | |  |  |  |  | ^x^ |  |  |  |  |  |  | | |  |  |  |  |  |  |  |  | | | | | | |  |  |  | ^M^ |  |
|  | Failure of some insurance companies to cover the cost of the vaccination (54.8%), and lack of adequate reimbursement (38.4%). |  |  |  |  |  |  |  |  |  |  |  |  |  |  |  |  |  | ^x^ |  |  |  |  |  |  |  |  |  |  |  |  |  |  |  |  |  |  |  |  |  |  |  |
|  | Vaccine expiring before use (54.1%) |  |  |  |  |  |  |  |  |  |  |  |  |  |  |  |  |  |  |  |  |  |  |  |  |  |  |  |  |  |  |  |  |  |  |  |  | ^x^ |  |  |  |  |
|  | Parental concerns about cost (53.3%) were also found to be perceived barriers. |  |  |  |  |  |  |  |  |  |  |  | ^x^ |  |  |  |  |  |  |  |  |  |  |  |  |  |  |  |  |  |  |  |  |  |  |  |  |  |  |  |  |  |
| Anup Srivastav *et al., l*  USA (43) | Pharmacists (not conducting routine vaccination assessments):  Lack of time or staff at the pharmacy to assess vaccines (69.4%).  Pharmacists (not recommending vaccinations to their adult patients):  Lack of time or staff at the pharmacy to recommend vaccines (65.2%)  Pharmacists (not administering vaccines):  Lack of staff to manage and administer vaccines (44.2%), Lack of necessary vaccine storage and handling Equipment and provisions (25.9%). |  | | | | | | | | |  |  |  |  |  |  |  |  |  |  |  | | |  |  |  |  |  |  |  |  | | | | | | | ^x^ |  |  |  |  |
|  | Inadequate vaccination expertise at the pharmacy (18.2%). |  |  |  |  |  |  |  |  |  |  |  |  |  |  |  |  |  |  |  |  |  |  |  |  |  |  |  |  |  |  |  |  |  |  |  |  |  | ^x^ |  |  |  |
|  | Pharmacists (not conducting routine vaccination assessments):  Vaccinations are not considered within the scope of practice (34.5%), and Vaccinations are not considered high priority by the practice (24.4%). |  |  |  |  |  |  |  |  |  |  |  |  |  |  |  |  |  |  |  |  |  |  |  |  |  |  |  |  | ^x^ |  |  |  |  |  |  |  |  |  |  |  | ^M^ |
|  | Pharmacists who reported not administering vaccines:  Inadequate reimbursement for vaccinations (28.8%) |  |  |  |  |  |  |  |  |  |  |  |  |  |  |  |  |  | ^x^ |  |  |  |  |  |  |  |  |  |  |  |  |  |  |  |  |  |  |  |  |  |  |  |
| Benjamin S. Teeter *et al.,* /  USA (44) | The requirement of a second refrigerator for vaccine storage and a secondary emergency and Lack of a private room for vaccine administration. |  | | | | | | | | |  |  |  |  |  |  |  |  |  |  |  | | |  |  |  |  |  |  |  |  | | | | | | | ^x^ |  |  |  |  |
|  | Misperceptions of the HPV vaccine, along with other vaccines, as barriers to education and provision of the vaccine and there is a lack of demand or knowledge for the vaccine.  Education and trust. |  |  |  |  |  |  |  |  |  |  |  |  |  | ^x^ |  |  |  |  |  |  |  |  |  |  |  |  |  |  |  |  |  |  |  |  |  |  |  |  |  |  |  |
| **Table 2. Barriers for Pharmacist-led Vaccination Services** | | **I. INNOVATION DOMAIN** | | | | | | | | | | | | **II. OUTER SETTING DOMAIN** | | | | | | | | | | **III. INNER SETTING DOMAIN** | | | | | | | | | | | | | | | | **IV.**  **INDIVIDUAL DOMAIN** | | |
| **Author/Country/Citation** | **Study reported Barriers** | **A. Source** | **B. Evidence-Base** | | **C. Relative Advantage** | | **D. Adaptability** | | **E. Trialability** | | **F. Complexity** | **G. Design** | **H. Cost** | **A. Critical Incidents** | **B. Local Attitudes** | **C. Local Conditions** | **D. Partnership & Connections** | **E. Policies & Laws** | **F. Financing** | **External pressure** | **G1. Societal Pressure** | **G2. Market Pressure** | **G3. Performance-Measurement Pressure** | **1. Physical Infrastructure** | **2.Information Technology Infrastructure** | **3. Work Infrastructure** | **B. Relational Connections** | **C. Communications** | **D. Culture** | **E. Tension for Change** | **F. Compatibility** | | **G. Relative Priority** | | **H. Incentive Systems** | | **I. Mission Alignment** | **J. Available resources** | **K. Access to Knowledge & Information** | **A. High-level Leaders*** | **H. Innovation Recipients** | **I. Innovation deliverers** |
| S. Suresh Madhavan *et al.,* /  USA (45) | Availability of time 5.63 ± 1.59  Availability of area with pharmacy to administer vaccines 5.11 ± 2.05 |  | | | | | | | | |  |  |  |  |  |  |  |  |  |  |  | | |  |  |  |  |  |  |  |  | | | | | | | ^x^ |  |  |  |  |
|  | Legal liability 5.61 ± 1.62 |  |  |  |  |  |  |  |  |  |  |  |  |  |  |  |  | ^x^ |  |  |  |  |  |  |  |  |  |  |  |  |  |  |  |  |  |  |  |  |  |  |  |  |
|  | Level of reimbursement 5.33 ± 1.52 |  |  |  |  |  |  |  |  |  |  |  |  |  |  |  |  |  | ^x^ |  |  |  |  |  |  |  |  |  |  |  |  |  |  |  |  |  |  |  |  |  |  |  |
|  | Support of local physicians 5.14 ± 2.6 |  |  |  |  |  |  |  |  |  |  |  |  |  | ^x^ |  |  |  |  |  |  |  |  |  |  |  |  |  |  |  |  |  |  |  |  |  |  |  |  |  |  |  |
| Sarah E. Kelling *et al.,* /  USA (46) | Time (23.5%), Space (19.4%), and Staff (16.3%). |  | | | | | | | | |  |  |  |  |  |  |  |  |  |  |  | | |  |  |  |  |  |  |  |  | | | | | | | ^x^ |  |  |  |  |
|  | There being no interest among the pharmacy staff (23.5%). |  |  |  |  |  |  |  |  |  |  |  |  |  |  |  |  |  |  |  |  | | |  |  |  |  |  |  |  |  |  |  |  |  |  |  |  |  |  |  | ^M^ |
|  | Patients not wanting vaccines (17.3%). |  |  |  |  |  |  |  |  |  |  |  |  |  | ^x^ |  |  |  |  |  |  | | |  |  |  |  |  |  |  |  |  |  |  |  |  |  |  |  |  | ^M^ |  |
|  | Doctors’ offices administering vaccines (29.6%). |  |  |  |  |  |  |  |  |  |  |  |  |  |  |  |  |  |  | ^x^ |  | | |  |  |  |  |  |  |  |  |  |  |  |  |  |  |  |  |  |  |  |
| Jean Rémi Valiquette *et al.,* /  Canada (47) | Lack of time (90%). |  | | | | | | | | |  |  |  |  |  |  |  |  |  |  |  | | |  |  |  |  |  |  |  |  | | | | | | | ^x^ |  |  |  |  |
|  | Lack of training (92%) |  |  |  |  |  |  |  |  |  |  |  |  |  |  |  |  |  |  |  |  | | |  |  |  |  |  |  |  |  |  |  |  |  |  |  |  | ^x^ |  |  |  |
|  | Fear of potential adverse events (14%) uneasiness with blood and bodily fluids (25%). |  |  |  |  |  |  |  |  |  |  |  |  |  |  |  |  |  |  |  |  | | |  |  |  |  |  |  |  |  |  |  |  |  |  |  |  |  |  |  |  |
|  | Fear of antagonizing other professionals (25%).  Lack of demand (25%) |  |  |  |  |  |  |  |  |  |  |  |  |  | ^x^ |  |  |  |  |  |  | | |  |  |  |  |  |  |  |  |  |  |  |  |  |  |  |  |  |  |  |
|  |  |  |  |  |  |  |  |  |  |  |  |  |  |  |  |  |  |  |  |  |  | | |  |  |  |  |  |  |  |  |  |  |  |  |  |  |  |  |  | ^N^ |  |
| Nicholas Edwards *et al.,* /  Canada (48) | Availability of pharmacy space; availability of support staff. |  | | | | | | | | |  |  |  |  |  |  |  |  |  |  |  | | |  |  |  |  |  |  |  |  | | | | | | | ^x^ |  |  |  |  |
|  | Current knowledge about vaccines; current training about indications/contraindications. |  |  |  |  |  |  |  |  |  |  |  |  |  |  |  |  |  |  |  |  |  |  |  |  |  |  |  |  |  |  |  |  |  |  |  |  |  | ^x^ |  |  | ^C^ |
|  | Legal liability. |  |  |  |  |  |  |  |  |  |  |  |  |  |  |  |  | ^x^ |  |  |  |  |  |  |  |  |  |  |  |  |  |  |  |  |  |  |  |  |  |  |  |  |
|  | Reimbursement of expenses/supplies. |  |  |  |  |  |  |  |  |  |  |  |  |  |  |  |  |  | ^x^ |  |  |  |  |  |  |  |  |  |  |  |  |  |  |  |  |  |  |  |  |  |  |  |
|  | Support from physicians. |  |  |  |  |  |  |  |  |  |  |  |  |  | ^x^ |  |  |  |  |  |  |  |  |  |  |  |  |  |  |  |  |  |  |  |  |  |  |  |  |  |  |  |
| Sandra Gerges  *et al.,* /  Canada (49) | Breeching physician territory |  | | | | | | | | |  |  |  |  | ^x^ |  |  |  |  |  |  | | |  |  |  |  |  |  |  |  | | | | | | |  |  |  |  |  |
|  |  |  |  |  |  |  |  |  |  |  |  |  |  |  |  |  |  |  |  |  |  |  |  |  |  |  |  |  |  |  |  |  |  |  |  |  |  |  |  |  |  |  |
|  | Immunizing children |  |  |  |  |  |  |  |  |  |  |  |  |  |  |  |  |  |  |  |  |  |  |  |  |  |  |  |  |  |  |  |  |  |  |  |  |  |  |  |  | ^c^ |
|  | Workload |  |  |  |  |  |  |  |  |  |  |  |  |  |  |  |  |  |  |  |  |  |  |  |  | ^x^ |  |  |  |  |  |  |  |  |  |  |  |  |  |  |  |  |
| Tessa J. Hastings *et al.,* / USA  (38) | Pharmacy resource |  | | | | | | | | |  |  |  |  |  |  |  |  |  |  |  | | |  |  |  |  |  |  |  |  | | | | | | | ^x^ |  |  |  |  |
|  | Internal support |  |  |  |  |  |  |  |  |  |  |  |  |  |  |  |  |  |  |  |  |  |  |  |  |  |  |  |  | ^x^ |  |  |  |  |  |  |  |  |  |  |  |  |
|  | Insurance coverage |  |  |  |  |  |  |  |  |  |  |  |  |  |  |  |  |  | ^x^ |  |  |  |  |  |  |  |  |  |  |  |  |  |  |  |  |  |  |  |  |  |  |  |
| Solome Tadele  *et al.,* (63) | Lack of authorization (94.1%). |  | | | | | | | | |  |  |  |  |  |  |  | ^x^ |  |  |  | | |  |  |  |  |  |  |  |  | | | | | | |  |  |  |  |  |
|  | Insufficient staff or resources for implementation (70%). |  |  |  |  |  |  |  |  |  |  |  |  |  |  |  |  |  |  |  |  |  |  |  |  |  |  |  |  |  |  |  |  |  |  |  |  | ^x^ |  |  |  |  |
|  | Cost and Time associated with professional development and training (71.4%) |  |  |  |  |  |  |  |  |  |  |  |  |  |  |  |  |  |  |  |  |  |  |  |  |  |  |  |  |  |  |  |  |  |  |  |  |  | ^x^ |  |  |  |

Covid-19: Coronavirus Disease 2019.

C: Capability.

N: Needs

M: Motivation.

HPV: human papilloma virous.

SEIPs: The Systems Engineering Initiative for Patient Safety

| **Table 3. Facilitators for Pharmacist-led Vaccination Services** | | **I. INNOVATION DOMAIN** | | | | | | | | | | | | **II. OUTER SETTING DOMAIN** | | | | | | | | | | **III. INNER SETTING DOMAIN** | | | | | | | | | | | | | | | | **IV.**  **INDIVIDUAL DOMAIN** | | |
| --- | --- | --- | --- | --- | --- | --- | --- | --- | --- | --- | --- | --- | --- | --- | --- | --- | --- | --- | --- | --- | --- | --- | --- | --- | --- | --- | --- | --- | --- | --- | --- | --- | --- | --- | --- | --- | --- | --- | --- | --- | --- | --- |
| **Author/**  **Country**  **/Citation** | **Study reported Facilitators** | **A. Source** | | **B. Evidence-Base** | | **C. Relative Advantage** | | **D. Adaptability** | | **E. Trialability** | **F. Complexity** | **G. Design** | **H. Cost** | **A. Critical Incidents** | **B. Local Attitudes** | **C. Local Conditions** | **D. Partnership & Connections** | **E. Policies & Laws** | **F. Financing** | **G. External Pressure** | **G1. Societal Pressure** | **G2. Market pressure** | **G3. Performance-Measurement Pressure** | **1. Physical Infrastructure** | **2.Information Technology Infrastructure** | **3. Work Infrastructure** | **B. Relational Connections** | **C. Communications** | **D. Culture** | **E. Tension for Change** | **F. Compatibility** | **G. Relative Priority** | | **H. Incentive Systems** | | | **I. Mission Alignment** | **J. Available resources** | **K. Access to Knowledge & Information** | **A. High-level Leaders*** | **H. Innovation Recipients** | **I. Innovation deliverers** |
| H Laetitia Hattingh *et al.,* /  Australia (65) | Offering the service via both appointment-booking and walk-in (no waiting time) systems provides convenience and flexibility to consumers. |  | | | | | | | | |  |  |  |  |  |  |  |  |  |  |  |  |  |  |  | **^x^** |  |  |  |  |  | | | | | | |  |  |  |  |  |
|  | Credibility of pharmacists |  |  |  |  |  |  |  |  |  |  |  |  |  |  |  |  |  |  |  |  |  |  |  |  |  |  |  |  |  |  |  |  |  |  |  |  |  |  |  |  | **^C^** |
|  | Pharmacists–client relationship |  |  |  |  |  |  |  |  |  |  |  |  |  | **^x^** |  |  |  |  |  |  |  |  |  |  |  |  |  |  |  |  |  |  |  |  |  |  |  |  |  |  | **^O^** |
| Susi Ari Kristina *et al.,* /Indonesia (60) | Vaccination by pharmacists, according to respondents, can make it easier for patients to obtain the COVID-19 vaccine (3.54±0.54). |  | | | | | | | | |  |  | | |  |  |  | **^x^** |  |  |  |  |  |  |  |  |  |  |  |  |  | | | | | | |  |  |  | **^O^** |  |
|  | If pharmacists help administer vaccines, it will build trust between pharmacists and patients (3.57±1.19) |  |  |  |  |  |  |  |  |  |  |  |  |  | **^x^** |  |  |  |  |  |  |  |  |  |  |  |  |  |  |  |  |  |  |  |  |  |  |  |  |  |  | **^O^** |
| Wei Chern Ang *et al.,* /  Malaysia (61) | More university education and training courses on vaccination administration for pharmacists (55.1 % strongly agreed, 41.1% agreed).  Continuous education and training workshops on vaccination (61.2% strongly agreed, 37% agreed) |  | | | | | | | | |  |  |  |  |  |  |  |  |  |  |  |  |  |  |  |  |  |  |  |  |  | | | | | | |  | **^x^** |  |  |  |
|  | Providing specific space for vaccination (49.8 % strongly agreed, 46.5% agreed).  Providing specific space to store vaccines (55.1 % strongly agreed, 42.3% agreed).  More pharmacists and staff in pharmacies to allocate time and provide an individual approach to patients for vaccination services (45.5 % strongly agreed, 50.2% agreed). |  |  |  |  |  |  |  |  |  |  |  |  |  |  |  |  |  |  |  |  |  |  |  |  |  |  |  |  |  |  |  |  |  |  |  |  | **^x^** |  |  |  |  |
|  | Reduce the workload of technical tasks for pharmacists (37.6 % strongly agreed, 49% agreed). |  |  |  |  |  |  |  |  |  |  |  |  |  |  |  |  |  |  |  |  |  |  |  |  | **^x^** |  |  |  |  |  |  |  |  |  |  |  |  |  |  |  |  |
|  | Cooperation between pharmacists and health professionals (61.4 % strongly agreed, 38% agreed).  Support of medical and nursing associations (55.1 % strongly agreed, 43.9% agreed). |  |  |  |  |  |  |  |  |  |  |  |  |  | **^x^** |  |  |  |  | | | | |  |  |  |  |  |  |  |  |  |  |  |  |  |  |  | |  |  | |
|  |  |  |  |  |  |  |  |  |  |  |  |  |  |  |  |  |  |  |  |  |  |  |  |  |  |  |  |  |  |  |  |  |  |  |  |  |  |  |  |  |  |  |
|  | Adequate reimbursement or remuneration of pharmacies (53.5% strongly agreed, 42.4 agreed). |  |  |  |  |  |  |  |  |  |  |  |  |  |  |  |  |  | **^x^** |  |  |  |  |  |  |  |  |  |  |  |  |  |  |  |  |  |  |  |  |  |  |  |
|  | Patients’ acceptance on the implementation of vaccination administered by pharmacists (44.7 % strongly agreed, 52.6% agreed). |  |  |  |  |  |  |  |  |  |  |  |  |  | **^x^** |  |  |  |  |  |  |  |  |  |  |  |  |  |  |  |  |  |  |  |  |  |  |  |  |  | **^M^** |  |
| Nikolaus Lindner *et al.,* /Austria (16) | Appropriate training (88.4%) |  | | | | | | | | |  |  |  |  |  |  |  |  |  |  |  |  |  |  |  |  |  |  |  |  |  | | | | | | |  | **^x^** |  |  |  |
|  | Liability insurance (78.2%) |  |  |  |  |  |  |  |  |  |  |  |  |  |  |  |  | **^x^** |  |  |  |  |  |  |  |  |  |  |  |  |  |  |  |  |  |  |  |  |  |  |  |  |
|  | Acceptance by patients (73.7%) |  |  |  |  |  |  |  |  |  |  |  |  |  | **^x^** |  |  |  |  |  |  |  |  |  |  |  |  |  |  |  |  |  |  |  |  |  |  |  |  |  | **^M^** |  |
|  | Acceptance by physicians (39.2%) |  |  |  |  |  |  |  |  |  |  |  |  |  | **^x^** |  |  |  |  |  |  |  |  |  |  |  |  |  |  |  |  |  |  |  |  |  |  |  |  |  |  |  |
|  | Financial remuneration (30.8%) |  |  |  |  |  |  |  |  |  |  |  |  |  |  |  |  |  | **^x^** |  |  |  |  |  |  |  |  |  |  |  |  |  |  |  |  |  |  |  |  |  |  |  |
| Piotr Merks *et al.,* /Poland (62) | Designate rooms for administering vaccinations in pharmacies (79.1%) |  | | | | | | | | |  |  |  |  |  |  |  |  |  |  |  |  |  |  |  |  |  |  |  |  |  | | | | | | | **^x^** |  |  |  |  |
|  | More university education and training courses for pharmacists in administering vaccinations are necessary (61.2%)  Obtaining a certificate confirming the ability to administer vaccinations (63.3 %) |  |  |  |  |  |  |  |  |  |  |  |  |  |  |  |  |  |  |  |  |  |  |  |  |  |  |  |  |  |  |  |  |  |  |  |  |  | **^x^** |  |  |  |
|  | Relevant remuneration or reimbursement for vaccination services. 84.8 % |  |  |  |  |  |  |  |  |  |  |  |  |  |  |  |  |  | **^x^** |  |  |  |  |  |  |  |  |  |  |  |  |  |  |  |  |  |  |  |  |  |  |  |
|  | Cooperation between pharmacists and healthcare centers was necessary (59.8%) |  |  |  |  |  |  |  |  |  |  |  |  |  | **^x^** |  |  |  |  |  |  |  |  |  |  |  |  |  |  |  |  |  |  |  |  |  |  |  |  |  |  |  |
| **Table 3. Facilitators for Pharmacist-led Vaccination Services** | | **I. INNOVATION DOMAIN** | | | | | | | | | | | | **II. OUTER SETTING DOMAIN** | | | | | | | | | | **III. INNER SETTING DOMAIN** | | | | | | | | | | | | | | | | **IV. INDIVIDUAL DOMAIN** | | |
| **Author/Country/Citation** | **Study reported Facilitators** | **A. Source** | **B. Evidence-Base** | | **C. Relative Advantage** | | **D. Adaptability** | | **E. Trialability** | | **F. Complexity** | **G. Design** | **H. Cost** | **A. Critical Incidents** | **B. Local Attitudes** | **C. Local Conditions** | **D. Partnership & Connections** | **E. Policies & Laws** | **F. Financing** | **G. External Pressure** | **G1. Societal Pressure** | **G2. Market pressure** | **G3. Performance-Measurement Pressure** | **1. Physical Infrastructure** | **2.Information Technology Infrastructure** | **3. Work Infrastructure** | **B. Relational Connections** | **C. Communications** | **D. Culture** | **E. Tension for Change** | **F. Compatibility** | **G. Relative Priority** | | **H. Incentive Systems** | | | **I. Mission Alignment** | **J. Available resources** | **K. Access to Knowledge & Information** | **A. High-level Leaders*** | **H. Innovation Recipients** | **I. Innovation deliverers** |
| Adam Pattison *et al.,* /UK (64) | Professional Recognition |  | | | | | | | | |  |  |  |  |  |  |  | **^x^** |  |  |  |  |  |  |  |  |  |  |  |  |  |  | |  | | |  |  |  |  |  | **^O^** |
|  | Access to Information |  |  |  |  |  |  |  |  |  |  |  |  |  |  |  |  |  |  |  |  |  |  |  | **^x^** |  |  |  |  |  |  |  |  |  |  |  |  |  |  |  |  |  |
|  | Social Responsibility |  |  |  |  |  |  |  |  |  |  |  |  |  |  |  |  |  |  |  |  |  |  |  |  |  |  |  |  |  |  |  |  |  |  |  |  |  |  |  |  | **^O^** |
| Mohammad B Nusair *et al.,* /Jordan  (55) | Restricting influenza vaccine sales to pharmacies would support the role of pharmacists as immunizers |  | | | | | | | | |  |  |  |  |  |  |  | **^x^** |  |  |  |  |  |  |  |  |  |  |  |  |  | | | | | | |  |  |  |  |  |
|  | Providing pharmacists with proper training on vaccine administration. |  |  |  |  |  |  |  |  |  |  |  |  |  |  |  |  |  |  |  |  |  |  |  |  |  |  |  |  |  |  |  |  |  |  |  |  |  | **^x^** |  |  |  |
|  | Role of health insurance companies |  |  |  |  |  |  |  |  |  |  |  |  |  |  |  |  |  | **^x^** |  |  |  |  |  |  |  |  |  |  |  |  |  |  |  |  |  |  |  |  |  |  |  |
| Mansour M. Alotaibi *et al.,* /Saudi Arabia (58) | People trust pharmacists as vaccine providers. |  | | | | | | | | |  |  |  |  | ^x^ |  |  |  |  |  |  |  |  |  |  |  |  |  |  |  |  | | | | | | |  |  |  |  | ^O^ |
| Dalal Youssef *et al.,* / Lebanon (14) | Patients demand (95%) |  | | | | | | | | |  |  |  |  |  |  |  |  |  |  |  |  |  |  |  |  |  |  |  |  |  | | | | | | |  |  |  | ^N^ |  |
|  | Support of health authorities (99.3 %)  Support from medical and nursing associations (81.6%) |  |  |  |  |  |  |  |  |  |  |  |  |  | ^x^ |  |  |  |  |  |  |  |  |  |  |  |  |  |  |  |  |  |  |  |  |  |  |  |  |  |  |  |
|  | Pharmacist interest (96.1%) |  |  |  |  |  |  |  |  |  |  |  |  |  |  |  |  |  |  |  |  |  |  |  |  |  |  |  |  |  |  |  |  |  |  |  |  |  |  |  |  | ^M^ |
|  | Continuous education and training (93.7%)  More university education and training on immunization administration for pharmacists (83.7%) |  |  |  |  |  |  |  |  |  |  |  |  |  |  |  |  |  |  |  |  |  |  |  |  |  |  |  |  |  |  |  |  |  |  |  |  |  | ^x^ |  |  |  |
|  | Financial reimbursement or adequate remuneration (84%) |  |  |  |  |  |  |  |  |  |  |  |  |  |  |  |  |  | ^x^ |  |  |  |  |  |  |  |  |  |  |  |  |  |  |  |  |  |  |  |  |  |  |  |
|  | Collaboration with medical clinics (79.1%) |  |  |  |  |  |  |  |  |  |  |  |  |  | ^x^ |  |  |  |  |  |  |  |  |  |  |  |  |  |  |  |  |  |  |  |  |  |  |  |  |  |  |  |
|  |  |  |  |  |  |  |  |  |  |  |  |  |  |  |  |  |  |  |  |  |  |  |  |  |  |  |  |  |  |  |  |  |  |  |  |  |  |  |  |  |  |  |
|  | Legal issues (82.8 %) |  |  |  |  |  |  |  |  |  |  |  |  |  |  |  |  | ^x^ |  |  |  |  |  |  |  |  |  |  |  |  |  |  |  |  |  |  |  |  |  |  |  |  |
| Bander Balkhi  *et al.,* /  Saudi Arabia  (59) | More university education and training on immunization administration for pharmacists.  Continuous education and training workshops on immunization |  | | | | | | | | |  |  |  |  |  |  |  |  |  |  |  |  |  |  |  |  |  |  |  |  |  | | | | | | |  | ^x^ |  |  |  |
|  | Financial reimbursement or adequate remuneration |  |  |  |  |  |  |  |  |  |  |  |  |  |  |  |  |  | ^x^ |  |  |  |  |  |  |  |  |  |  |  |  |  |  |  |  |  |  |  |  |  |  |  |
|  | Patients’ demand |  |  |  |  |  |  |  |  |  |  |  |  |  | ^x^ |  |  |  |  |  |  |  |  |  |  |  |  |  |  |  |  |  |  |  |  |  |  |  |  |  | ^N^ |  |
|  | Providing a specific area for immunization in the pharmacy |  |  |  |  |  |  |  |  |  |  |  |  |  |  |  |  |  |  |  |  |  |  |  |  |  |  |  |  |  |  |  |  |  |  |  |  | ^x^ |  |  |  |  |
|  | Collaboration with medical clinic |  |  |  |  |  |  |  |  |  |  |  |  |  | ^x^ |  |  |  |  |  |  |  |  |  |  |  |  |  |  |  |  |  |  |  |  |  |  |  |  |  |  |  |
|  | Support of medical and nursing associations |  |  |  |  |  |  |  |  |  |  |  |  |  | ^x^ |  |  |  |  |  |  |  |  |  |  |  |  |  |  |  |  |  |  |  |  |  |  |  |  |  |  |  |
|  | Pharmacist’s specialty interest |  |  |  |  |  |  |  |  |  |  |  |  |  |  |  |  |  |  |  |  |  |  |  |  |  |  |  |  |  |  |  |  |  |  |  |  |  |  |  |  | ^C^ |
| Tessa J. Hastings *et al.,* / USA (38) | External support after training program implementation p value (0.023) |  | | | | | | | | |  |  |  |  |  |  |  |  |  |  |  |  |  |  |  |  |  |  |  |  |  | | | | | | |  | ^x^ |  |  |  |
| Jessica  *et al., l*  USA  (54) | Education/promotion to public and patients, adolescent (33%) adult (40%). |  | | | | | | | | |  |  |  |  |  |  |  |  |  |  |  |  |  |  |  |  |  |  |  |  |  | | | | | | |  |  |  |  | ^C^ |
|  | Staff time and participation, adult (43%). |  |  |  |  |  |  |  |  |  |  |  |  |  |  |  |  |  |  |  |  |  |  |  |  |  |  |  |  |  |  |  |  |  |  |  |  | ^x^ |  |  |  |  |
|  | State legislative authority to provide vaccine, adolescent (51%).  Clear guidelines from corporate management/protocol, adolescent (31%) adult (38%). |  |  |  |  |  |  |  |  |  |  |  |  |  |  |  |  | ^x^ |  |  |  |  |  |  |  |  |  |  |  |  |  |  |  |  |  |  |  |  |  |  |  |  |
| Daniyal *et al.,l* Canada (51) | Increasing vaccination rates  Reducing risk of infection |  | | | | | | | | |  |  |  |  |  |  |  |  |  |  |  |  |  |  |  |  |  |  |  |  |  | | | | | | |  |  |  | ^N^ |  |
| Kevin A. *et al.,* /  USA (36) | Immunization training program would be beneficial (83.7%) |  | | | | | | | | |  |  |  |  |  |  |  |  |  |  |  |  |  |  |  |  |  |  |  |  |  | | | | | | |  | ^x^ |  |  |  |
|  | Pharmacists replied that they were comfortable with administering immunizations (44.2%). Pharmacists felt they were able to handle potential problems, such as needle sticks, during training (53.5%) |  |  |  |  |  |  |  |  |  |  |  |  |  |  |  |  |  |  |  |  |  |  |  |  |  |  |  |  |  |  |  |  |  |  |  |  |  |  |  |  | ^c^ |
|  | Comfortable with the billing procedures for immunizations (60.5%) |  |  |  |  |  |  |  |  |  |  |  |  |  |  |  |  |  | ^x^ |  |  |  |  |  |  |  |  |  |  |  |  |  |  |  |  |  |  |  |  |  |  |  |
| **Table 3. Facilitators for Pharmacist-led Vaccination Services** | | **I. INNOVATION DOMAIN** | | | | | | | | | | | | **II. OUTER SETTING DOMAIN** | | | | | | | | | | **III. INNER SETTING DOMAIN** | | | | | | | | | | | | | | | | **IV. INDIVIDUAL DOMAIN** | | |
| **Author/Country/Citation** | **Study reported Facilitators** | **A. Source** | **B. Evidence-Base** | | **C. Relative Advantage** | | **D. Adaptability** | | **E. Trialability** | | **F. Complexity** | **G. Design** | **H. Cost** | **A. Critical Incidents** | **B. Local Attitudes** | **C. Local Conditions** | **D. Partnership & Connections** | **E. Policies & Laws** | **F. Financing** | **G. External Pressure** | **G1. Societal Pressure** | **G2. Market pressure** | **G3. Performance-Measurement Pressure** | **1. Physical Infrastructure** | **2.Information Technology Infrastructure** | **3. Work Infrastructure** | **B. Relational Connections** | **C. Communications** | **D. Culture** | **E. Tension for Change** | **F. Compatibility** | | **G. Relative Priority** | | **H. Incentive Systems** | **I. Mission Alignment** | | **J. Available resources** | **K. Access to Knowledge & Information** | **A. High-level Leaders*** | **H. Innovation Recipients** | **I. Innovation deliverers** |
| Maurice N. Tran  *et al.,* /  USA (37) | Patient-centered education on immunizations was perceived as an enabler. |  | | | | | | | | |  |  |  |  |  |  |  |  |  |  |  |  |  |  |  |  |  |  |  |  |  | | | | | | |  |  |  |  | ^C^ |
|  | Pharmacy’s physical layout was perceived to facilitate immunization outreach efforts. |  |  |  |  |  |  |  |  |  |  |  |  |  |  |  |  |  |  |  |  |  |  | ^x^ |  |  |  |  |  |  |  |  |  |  |  |  |  |  |  |  |  |  |
|  | Electronically access a patient’s consolidated vaccination record through the immunization platform as a facilitator to proactively identifying vaccination needs. |  |  |  |  |  |  |  |  |  |  |  |  |  |  |  |  |  |  |  |  |  |  |  | ^x^ |  |  |  |  |  |  |  |  |  |  |  |  |  |  |  |  |  |
| Benjamin S. Teeter et al., /USA (44) | Intervention characteristics:  Since vaccinations were already provided in the participating pharmacies, many expressed that there was difference between what they are currently doing and what would be required if were asked to increase the HPV vaccina  Pharmacists and pharmacy technicians reported being knowledgeable about the HPV vaccine and pharmacists were confident in their ability to provide the vaccine and counsel patients.  Pharmacy staff interviewed had positive attitudes toward administering vaccine. |  | | | | | | | | |  |  |  |  |  |  |  |  |  |  |  |  |  |  |  |  |  |  |  |  |  | | | | | | |  |  |  |  | ^c^ |
|  |  |  |  |  |  |  |  |  |  |  |  |  |  |  |  |  |  |  |  |  |  |  |  |  |  |  |  |  |  |  |  |  |  |  |  |  |  |  |  |  |  |  |
| Jean Rémi Valiquette et al., / Canada  (47) | Increased immunization training (95%). |  | | | | | | | | |  |  |  |  |  |  |  |  |  |  |  |  |  |  |  |  |  |  |  |  |  | | | | | | |  | ^x^ |  |  |  |
|  | Pharmacists’ personal interest (69 %). |  |  |  |  |  |  |  |  |  |  |  |  |  |  |  |  |  |  |  |  |  |  |  |  |  |  |  |  |  |  |  |  |  |  |  |  |  |  |  |  | ^M^ |
|  | Adequate remuneration (92%). |  |  |  |  |  |  |  |  |  |  |  |  |  |  |  |  |  | ^x^ |  |  |  |  |  |  |  |  |  |  |  |  |  |  |  |  |  |  |  |  |  |  |  |
| Sandra Gerges et al., /  Canada  (49) | Strengthen relationships with patients and increase patient satisfaction.  Increase patients’ trust  Increased convenience to patients |  | | | | | | | | |  |  |  |  | ^x^ |  |  |  |  |  |  |  |  |  |  |  |  |  |  |  |  | | | | | | |  |  |  |  | ^O^ |

Covid-19: Coronavirus Disease 2019.

C: Capability.

N: Needs

M: Motivation.

O: opportunity.

HPV: human papilloma virous.
